# Comparative Transcriptome Analysis of the Necrotrophic Fungus *Ascochyta rabiei* during Oxidative Stress: Insight for Fungal Survival in the Host Plant

**DOI:** 10.1371/journal.pone.0033128

**Published:** 2012-03-12

**Authors:** Kunal Singh, Shadab Nizam, Manisha Sinha, Praveen K. Verma

**Affiliations:** Plant Immunity Laboratory, National Institute of Plant Genome Research, Aruna Asaf Ali Marg, New Delhi, India; Nanjing Agricultural University, China

## Abstract

Localized cell death, known as the hypersensitive response (HR), is an important defense mechanism for neutralizing phytopathogens. The hallmark of the HR is an oxidative burst produced by the host plant. We aimed to identify genes of the necrotrophic chickpea blight fungus *Ascochyta rabiei* that are involved in counteracting oxidative stress. A subtractive cDNA library was constructed after menadione treatment, which resulted in the isolation of 128 unigenes. A reverse northern blot was used to compare transcript profiles after H_2_O_2_, menadione and sodium nitroprusside treatments. A total of 70 unigenes were found to be upregulated by more than two-fold following menadione treatment at different time intervals. A large number of genes not previously associated with oxidative stress were identified, along with many stress-responsive genes. Differential expression patterns of several genes were validated by quantitative real-time PCR (qRT-PCR) and northern blotting. *In planta* qRT-PCR of several selected genes also showed differential expression patterns during infection and disease progression. These data shed light on the molecular responses of the phytopathogen *A. rabiei* to overcome oxidative and nitrosative stresses and advance the understanding of necrotrophic fungal pathogen survival mechanisms.

## Introduction

The capability of pathogenic fungi to cause disease requires competence to survive in the host. Pathogen survival in the host is in turn dependent on evading or suppressing the host's immune responses. Early responses towards attempted pathogen attack of plants and animals are often accompanied by a coordinate activation of programmed cell death (PCD) and defense mechanisms [1]. In plants, this response is termed the Hypersensitive Response (HR) and is orchestrated by an oxidative burst, which induces localized cell death at the infection site [2], [3]. This oxidative burst consists of a biphasic production of Reactive Oxygen Species (ROS) at the site of attempted pathogen invasion [4]–[5]. The HR is an important element of the defense strategy that plants employ against biotrophic pathogens, which derive nutrition from living tissues [6]. In contrast, necrotrophic fungi obtain nutrients exclusively from dead tissues and produce toxins as well as cell wall-degrading enzymes that kill host cells prior to invasion [7]. The HR produced by the host is reported to facilitate colonization by necrotrophs such as *Botrytis cinerea* and *Sclerotinia sclerotiorum* [8]. Consequently, necrotrophs are able to exploit host defense mechanisms, enlarge the infection field and colonize host tissues [8]. The mechanism by which necrotrophs exploit the HR for growth is largely unknown. To thrive within the oxidative environment of necrotic tissues, pathogenic fungi have evolved multiple defense systems, both enzymatic and non-enzymatic [9], [10]. The necrotroph *B. cinerea* has been reported to have an array of enzymes including catalase and superoxide dismutase that protect it against an environment rich in ROS [9], [11]. Targeted gene replacement of *Magnaporthe oryzae* catalase *CATB* gene led to compromised pathogen fitness and reduced pathogenicity [12]. Recently the *M. oryzae* transcription factor MoAtf1 was found to be necessary for complete virulence and oxidative stress response of the fungus [13]. An important homolog of the yeast transcription factor Yap1 from *Cochliobolus heterostrophus* and *Ustilago maydis* was reported to regulate oxidative stress as well as virulence [14]–[15]. These studies suggest that the genes involved in ROS detoxification and oxidative-stress response are vital for fungal survival and pathogenesis. ROS also plays an important role during mutualistic interactions between the fungal endophyte *Epichloe festucae* and its grass host *Lolium perenne* [16].

In plants, nitric oxide (NO) is an important signaling molecule that regulates a number of critical signal transduction pathways, including the defense response [17]. NO, together with ROS, has emerged as a major participant in the HR and plant cell death [18]. Recent work on plant defense mechanisms has indicated that establishing and initiating the HR requires the correct relative levels of both NO and ROS to be produced in the host [19], [20]. Crosstalk between ROS and RNS (reactive nitrogen species) during the HR is required for the host resistance response [4]. The human pathogenic fungus *Cryptococcus neoformans* counteracts cytotoxic nitrosative stress with the help of enzymes, such as flavohemoglobin denitrosylase and S-nitrosoglutathione reductase, which were shown to promote fungal virulence [21]. Similarly, a gene encoding flavohemoglobin (*BcFHG1*) was shown to be essential for NO detoxification and provide protection against nitrosative conditions in the necrotrophic fungus *B. cinerea* [22]. Therefore, the relative sensitivity of fungal pathogens to oxidative and nitrosative stress depends on the effectiveness of their own ROS and RNS detoxification machinery.

Transcript profiling is an important strategy for studying the expression of large gene sets modulated against a particular condition. The DNA array strategy has been applied in fungi to investigate the transcript pattern at the time of spore germination [23], appressorium formation [24] and pathogenesis [25]. Furthermore, most studies have focused on various aspects of development and infection processes [26] although a few reports have described transcriptional changes due to oxidative stress [27].

The necrotrophic fungus *Ascochyta rabiei* (Pass.) Lab. (teleomorph: *Didymella rabiei*) causes Ascochyta blight (AB) disease of the chickpea (*Cicer arietinum* L.), which is the second most important food legume worldwide in terms of productivity [28], and results in severe losses in grain yield. There is a high degree of variation in resistance among chickpea cultivars, but complete resistance to *A. rabiei* has not been observed [29]. *A. rabiei* causes typical circular necrotic lesions on all above ground plant parts. The plant dies when the main stem is girdled at the collar region [30], [31]. The generation of a rapid oxidative burst accompanied by the HR was reported to occur in the chickpea-*Ascochyta* interaction in tolerant cultivars [32], [33]. ROS accumulation and subsequent HR were also observed both in moderately resistant and susceptible chickpea cultivars in the course of *Ascochyta* infection [34].

In this study, we aimed to identify oxidative stress-induced genes of the phytopathogenic fungus *A. rabiei*. To isolate such genes, a subtractive cDNA library was constructed from menadione-treated *A. rabiei* cultures. In addition to well known stress-responsive genes, a large number of genes not previously associated with oxidative stress were identified. The expression patterns of the isolated genes were analyzed in response to both oxidative and nitrosative stress conditions. *In planta* expression studies of several selected genes revealed high expression during infection. This study provides new insights about survival of *A. rabiei* against oxidative and nitrosative stresses.

## Materials and Methods

### Fungal isolates and stress treatments

Pure cultures of *Ascochyta rabiei* (Delhi isolate, D-11) were obtained from IARI, New Delhi. Cultures were grown on Potato Dextrose Agar (PDA; Difco Laboratories, USA) or in Potato Dextrose Broth (PDB; Difco) with shaking at 120 rpm for four days at 22°C in the dark. For exogenous stress treatments, fungal spore suspensions (1×10^3^ spores/ml) were grown for four days at 120 rpm and subjected to treatment with menadione (250 µM, Sigma-Aldrich, USA), hydrogen peroxide (H_2_O_2_; 5 mM, Sigma-Aldrich) or sodium nitroprusside (SNP; 500 µM, Sigma-Aldrich). Fungal cultures were harvested at 0.5, 1, and 3 h after treatment with menadione, and 1 h after H_2_O_2_ and SNP treatments. Mock-treated samples with the respective solvents were used as controls. One month old chickpea plants (Pusa-362) were inoculated with *A. rabiei* spore suspensions (1±10^6^ spores/ml). Infected stems and leaves were collected 1, 3 and 6 d post-inoculation (dpi).

### Histochemical detection of ROS and microscopy

The chickpea leaves and stem peels were inoculated with WT and DsRed-expressing *A. rabiei* [31]. Two days after infection, tissues were stained with 3, 3′-diaminobenzidine (DAB) using the DAB-Black kit (Invitrogen, Paisley, UK) according to the manufacturer's instructions. Brownish-black colored polymerization products resulting from the reaction of DAB with ROS were visualized using a Zeiss Axio Examiner microscope with differential interference contrast optics. DsRed fluorescence was detected with standard filters for rhodamine (excitation 546/12, beam splitter 560, emission 607/80 nm; Zeiss). Images were obtained with a CCD camera.

### Assessment of optimal menadione dose

*Ascochyta* spore suspension (1×10^3^ spores/ml) cultures were grown with shaking for four days in PDB as described above with different menadione concentrations (0.1, 0.25, 1 and 20 mM) added before the cultures were immediately transferred back to the shaker. After 24 h of treatment, the mycelial ball diameters were assessed. In order to measure the fungal biomass dry weight, mycelial balls were filtered through Whatman filter paper No. 1 and dried at 50°C for 72 h in an air incubator. Three independent experiments were performed each time with three sets of technical replicates and respective data set means (± SE) were estimated from the above cultures.

### Isolation of RNA and construction of subtracted cDNA library

To construct the subtracted cDNA library, total RNA was isolated from *A. rabiei* using the TRIzol® reagent (Invitrogen, USA). Poly A^+^ RNA was purified using an mRNA isolation kit (Roche, Germany) according to the manufacturer's protocol. A forward Suppression Subtractive Hybridization (SSH) was performed using a PCR-Select™ cDNA Subtraction Kit (BD Biosciences, USA) according to the manufacturer's protocol. The mRNA isolated from the 1 h menadione-treated sample was used as the ‘tester’, where as the mock-treated sample was used as the ‘driver’ for subtraction. The enriched differentially expressed cDNAs were cloned into the pDrive U/A Cloning Vector (Qiagen, Germany). Recombinant plasmids were further sequenced using the Big Dye Terminator™ kit version 3.0 (Applied Biosystems, USA) and examined with the 3700 ABI Prizm 96 capillary sequence analyzer. All sequences were screened for homology in the GenBank database using BLASTx and tBLASTx (http://www.ncbi.nlm.nih.gov/BLAST/). Sequences were submitted to GenBank and the assigned accession numbers are listed in Table S1.

### cDNA macroarray and data analysis

Individual clones of the subtracted cDNA library were amplified, purified, and denatured by adding an equal volume of 0.6 M NaOH. Equal volumes of each denatured PCR product (about 100 ng) were spotted on Hybond™ N membranes (Amersham, USA) using a 96-well dot-blot apparatus (Bio-Rad, USA). In addition, PCR products of *A. rabiei* Elongation factor 1 alpha (*ArEf1a*) cDNA using primers EF1a-F (5′-TCGGTGTCAAGCAGCTCATC-3′) and EF1a-R (5′-AAGCCTCAACGCACATGG-3′) and the neomycin phosphotransferase (*NPTII*) gene from the binary vector pBI121 (Accession No. AF485783.1) using primers NPTII-F (5′-TGCTCGACGTTGTCACTGAAG-3′) and NPTII-R (5′-GTCAAGAAGGCGATAGAAGGC-3′) were spotted as an internal and negative control, respectively. The membranes were neutralized with neutralization buffer (0.5 M Tris-HCl, pH 7.4, 1.5 M NaCl) for 3 min, washed with 2× SSC and immobilized with UV cross-linker (Stratagene, USA). Probes were prepared for DNA array hybridization by first-strand reverse transcription (Primescript™ RT, BD Biosciences, USA) with 1 µg mRNA isolated from menadione, H_2_O_2_ and SNP-treated samples and labeled with α^32^P-dCTP (10 µCi µl^−1^; 3,000 Ci mmol^−1^). Radiolabeled cDNAs were purified on a Sephadex G-50 column (GE Healthcare, Sweden), suspended in pre-hybridization buffer (7% SDS, 0.3 M Sodium phosphate buffer pH 7.4, 1 mM EDTA) and hybridized at 60°C overnight. The membranes were initially washed three times with washing buffer (1× SSC, 1% SDS, 20 min each at 60°C). Autoradiographs were scanned with a Fluor-S-Multi-imager (FSMI; Bio-Rad) to acquire images. Detection and quantification of signals representing the hybridized cDNAs were performed using Quantity One™ software version 4.2.3 (Bio-Rad) and signal intensities were analyzed by subtracting the background. A total of six replicates representing three biological replicates were analyzed for all experiments. *ArEf1a* cDNA was used as an internal control where the subtracted density value was used for normalization. Differential screening and expression pattern data were generated as the mean (±SD) of expression ratios for all independent experiments. A paired Student's *t*-test on log_2_-transformed data was applied to determine whether statistical differences between the expression ratios of each treatment and control pair were evident. Genes that were significantly different from controls in any of the treatments were selected and presented. The following two criteria were chosen to demarcate differentially expressing genes based on previous reports [35]: (a) a greater than two-fold induction level; and (b) a P<0.05 level of significance as determined by a t-test for each experiment, and through analysis of variance (ANOVA) [36]. ANOVA was performed for each clone and the significance of differences in expression patterns was tested with a high stringency threshold *P* value of 0.01 using InStat version 3.1 software (GraphPad). Expression profiles of inducible cDNAs were also analyzed by clustering performed by Self Organizing Tree Algorithm (SOTA) using average linkage by TIGR Multiple Experiment Viewer version 3.0 (available at http://www.tigr.org/software/tm4/menu/TM4).

### Northern hybridization

Ten micrograms of total RNA from control and treated samples were fractionated in a 1.2% agarose gel containing formaldehyde and transferred onto a positively charged Hybond™ N^+^ membrane (Amersham) according to Sambrook and Russell [37]. Equal loading and lane transfer was verified by membrane staining with methylene blue (0.02%). PCR-amplified individual cDNA fragments (with primers corresponding to adaptor 1 and 2R, provided in the SSH kit) were purified from agarose gels. In addition, β-tubulin TUB-F (5′-CATCTCCGGCGAGCATGGC-3′) and TUB-R (5′-CCAGTTGTTACCAGCACCAG-3′) was amplified and purified from the agarose gel. Probes were labeled with α^32^P-dCTP using the NEBlot® kit (New England Biolabs, USA) according to the manufacturer's instructions and purified as described above. Hybridization and high stringency washing was carried out using standard protocols [37]. Development and scanning of autoradiographs were carried out as previously described.

### Quantitative real-time PCR

Total RNA was extracted as described above and treated with DNase I (Promega, USA) according to manufacturer's protocol to remove any contaminating genomic DNA. First-strand cDNA was synthesized with 1 µg of total RNA primed with Oligo-dT using NovaScript III RNase H minus Reverse Transcriptase (Life Technologies, India) as per the instructions in the manual. The primers were designed using Primer Express® (version 3.0) software (Applied Biosystems) with the default parameters. The primers used for quantitative RT-PCR are shown in Table S2. Real-Time PCR (RT-PCR) was carried out in 96-well plates on a 7900HT Sequence Detection System using Sequence Detection Systems Software version 2.3 (Applied Biosystems) and Power SYBR Green PCR Master Mix (Applied Biosystems) in a final volume of 20 µl. The default cycling program was used with the following cycling conditions: 2 min at 50°C, 10 min at 95°C, and 40 cycles of 15 s at 95°C and 1 min at 60°C. Each experiment was performed with three replicates. The primer pair specificity was visualized by dissociation curve monitoring and agarose gel electrophoresis. The gene encoding ArEf1*a* was used as the gene for calibration in all experiments. Expression ratios were calculated from cycle threshold values using the 2^−ΔΔCT^ method.

## Results

### Menadione induces oxidative stress in *A. rabiei* broth culture

The Diaminobenzidine (DAB) staining and microscopic studies in the susceptible chickpea cultivar, Pusa 362 showed that intracellular ROS was generated after *A. rabiei* infection (Fig. S1). To isolate oxidative stress induced genes, menadione was used to generate ROS in broth cultures of *A. rabiei*. Menadione produces superoxide anion (O_2_^−^), which readily dismutates to H_2_O_2_ or combines with NO to form a strong oxidant peroxynitrite [38], [39]. Molecular, pharmacological and genetic studies also support the hypothesis that the primary source of ROS during HR is O_2_^−^ generated by plant NADPH oxidase [2]. Since previous studies showed the extremely toxic effect of menadione on certain filamentous fungi [40], we, therefore optimized the menadione concentration in the *A. rabiei* broth cultures to generate non-lethal doses of oxidative stress. The measurement of fungal dry weight and mycelia ball size was carried out after 24 h incubation. Both parameters suggested that a 250 µM menadione concentration could induce oxidative stress in the fungus without imposing high toxic effects (Fig. S2). At this menadione concentration, the mycelial growth returned to normal levels 48 h after treatment.

### Subtractive library identifies many unique and stress responsive genes

To study the early molecular responses of *A. rabiei* against oxidative stress, a forward subtractive cDNA library was constructed using the Suppression Subtractive Hybridization (SSH) strategy with the 1 h menadione-treated samples. The SSH strategy enriches the less abundant transcripts by normalizing and amplifying the subtracted cDNAs [41]. As a result, 756 recombinant clones were obtained that contained cDNA fragments ranging from 138 to 1024 bp with an average clone size of ∼350 bp. Annotation, screening and sequence analysis of these clones identified 128 independent sequences (unigenes) by similarity searches against GenBank databases (NCBI). Sequence analysis revealed that out of the 128 unigenes, 28.13% were present as a single copy, 13.28% were in duplicates and 58.59% were present three or more times in the library. Out of the 128 sequences, 13 (10.15%) showed no homology with known sequences and were classified as ‘no hits’. Seven sequences with high *E*-values were also considered as ‘no hits’. Two genes were also found with *P* values <0.05 after conducting a test of significance (*t*-test) and were eliminated from analysis. All of the remaining 106 genes were selected and used for further analysis.

**Figure 1:**
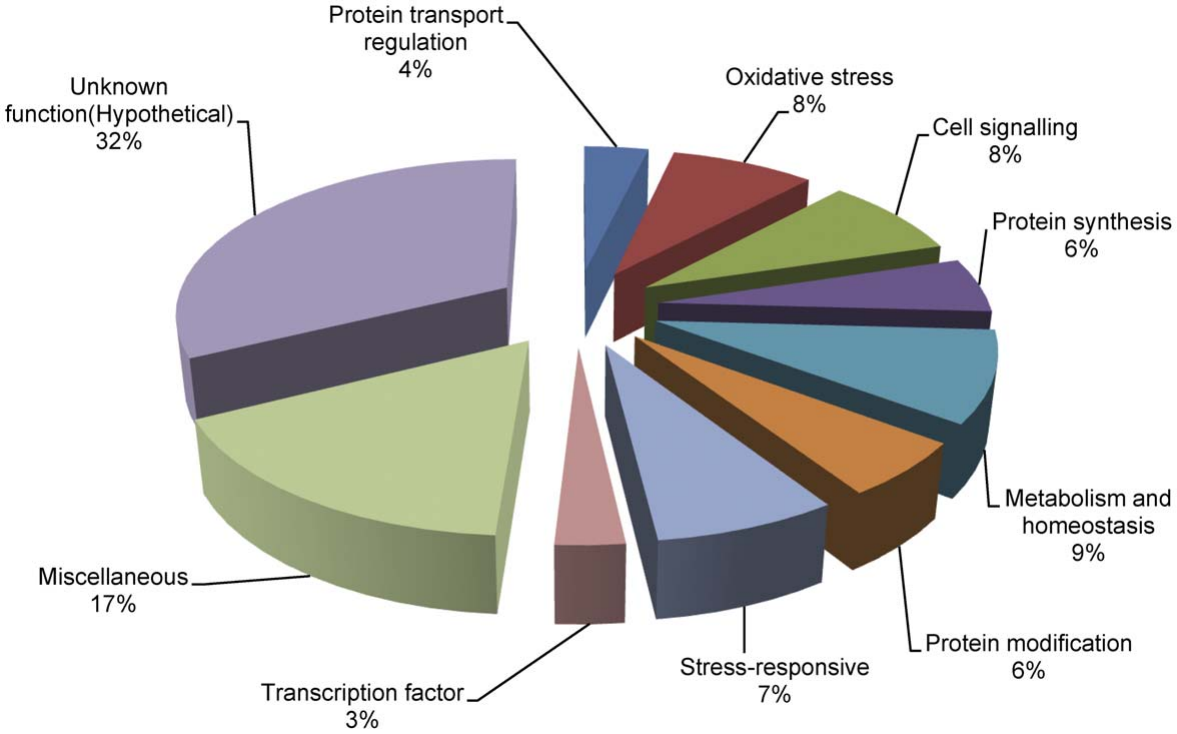
Functional cataloging of menadione-responsive genes. Genes were assigned a putative function based on their homology and classified on the basis of their functions.

The genes were classified into 10 functional categories based on their potential cellular function (Table S1; Fig. 1). Major functional categories corresponded to genes involved in stress including stress response (7%), protein modification (6%) and oxidative stress (8%). Other significant categories included protein transport (4%), protein synthesis (6%), metabolism and homeostasis (9%), miscellaneous (18%), cell signaling (8%), transcription (3%) and proteins of unknown function (31%). When the roles of gene products had overlapping functional categories they were classified according to their most probable role during oxidative stress. Genes known to be involved in ROS detoxification were well-represented in the library and included catalase (CAT), alternative oxidase, superoxide dismutase (SOD) and thioredoxin (TRX). Other genes with well-established functions during various stresses such as ubiquitin and glyceraldehyde 3-phosphate dehydrogenase (GAPDH) were categorized under stress responsive genes. Among the genes from other categories, export control protein CHS7-like (protein transport), serine/threonine phosphatase PP2A (PP2A, signal transduction), myo-inositol phosphate synthase (metabolism) and opsin (miscellaneous) were previously reported to be involved in the stress responses. Genes involved in fatty acid synthesis/metabolism were represented by ATP citrate synthase, fatty acid synthase subunit beta dehydratase and acyl-CoA desaturase.

### Expression kinetics after menadione-treatment identified several upregulated genes

**Figure 2:**
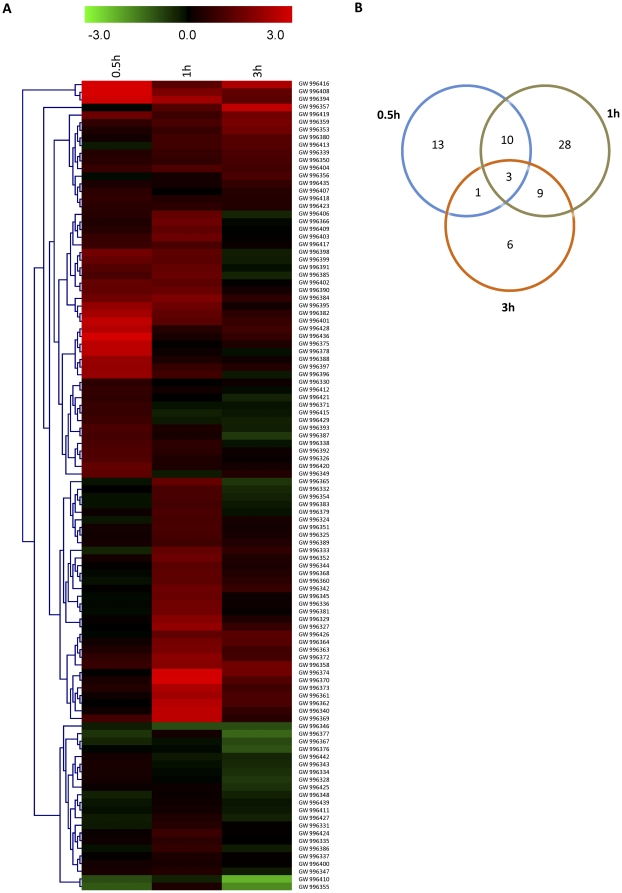
Comparison of gene expression after menadione treatment at different time intervals. (a) Heat map of hierarchical clustering performed for selected genes illustrates differential induction patterns after menadione-treatment for 0.5 h, 1 h and 3 h. (b) The Venn diagram represents the distribution of transcripts that was significantly induced (>2-fold) at different time intervals.

A macroarray analysis was performed to determine the early induction kinetics of all the unigenes. Samples were collected at 0.5, 1, and 3 h after 0.25 mM menadione treatment, as described in ‘Materials and Methods’. Since there was no sequence available in the database for any *A. rabiei* house-keeping genes, we amplified the cDNA sequences of elongation factor and tubulin using primers based on their conserved sequences. The constitutive expression of these genes during oxidative stress was confirmed by northern blot analysis (Fig. S3). On this basis, EF1a was used as an internal control during array hybridization experiments. The densitometry data obtained by macroarray analysis was statistically analyzed and used for generation of heat-map and cluster analysis (Fig. 2a). To reduce the noise level, expression ratios obtained by macroarray were log_2_ transformed. Genes were considered to be induced when the expression ratio increased by more than two-fold. Our results revealed that 27 genes were induced after 0.5 h treatment and increased to 51 genes after 1 h treatment (Fig. 2b), which substantiates that the library was indeed enriched with genes induced early on during oxidative stress. The number of genes with high expression was reduced to 19 at the 3 h time point and altogether a total of 70 genes were found to be upregulated. The expression results of fold-induction at different time-points are summarized in Table S1. A few genes showed early biphasic induction (*Ar93* and *Ar96*). A set of six genes (*Ar16*, *Ar27*, *Ar30*, *Ar33*, *Ar57* and *Ar90*) showed induction 1 h after menadione treatment, but their expression was increased by two-fold only at 3 h post-treatment. A total of 36 genes were found to be not induced or below the cut-off value at all three time points, indicating that these genes either were induced late after treatment or to levels below the two-fold cut-off value.

### Comparative expression analysis in response to menadione, H_2_O_2_ and SNP revealed that several genes have similar expression patterns

**Figure 3:**
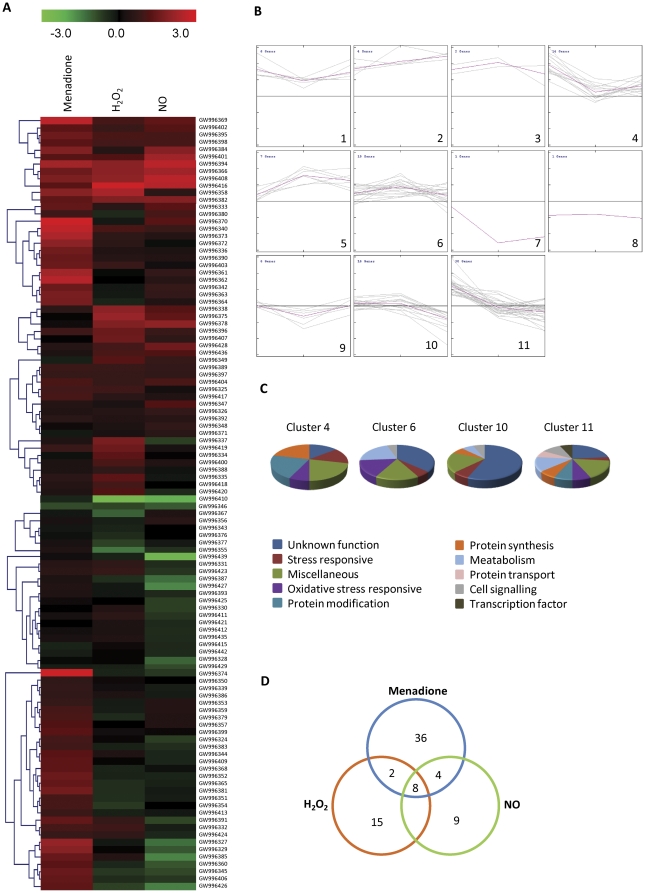
Clustering analysis of menadione-responsive gene expression profiles. (a) SOTA cluster tree of selected genes illustrates differential induction patterns after menadione, H_2_O_2_ and NO treatments. Accession numbers provided by NCBI are given in the heat map and the corresponding gene names are listed in Table S1. (b) The 106 genes were grouped into 11 clusters based on their expression profiles. The expression profile of each individual gene in the cluster is depicted by grey lines, while the mean expression profile is marked in pink for each cluster. The number of genes in each cluster is given in the left upper corner and the cluster number in the right lower corner. (c) Functional cataloging of the genes present in different clusters (clusters with n>10 were taken into consideration). (d) The Venn diagram represents the distribution of transcripts that were significantly induced (>2 fold) in macroarray analysis after menadione, H_2_O_2_ and NO treatments.

To achieve a comprehensive overview of the expression pattern of genes that were co-expressed during menadione, H_2_O_2_, and SNP treatment, SOTA clustering was performed by analyzing macroarrays of samples collected 1 h after each treatment (Fig. 3a, b). SOTA analysis yielded 11 clusters and those with n>10 were selected for additional studies of the expression patterns of functionally similar genes (Fig. 3b, c). The details of each cluster are provided in Table S3. The maximum number of genes were grouped into cluster 11 (30 genes), which was comprised of genes having high expression during menadione and H_2_O_2_ treatments and represented all of the functional categories. The second major group was cluster 6, which contained 19 genes that are involved in oxidative stress and lipid metabolism. Genes from miscellaneous and unknown function categories were represented in almost all the clusters, which may be due to the heterogeneous composition of these categories and their high representation in the library (Table S1). In addition, expression was compared on the basis of induction level (fold-induction), and fifty genes showed greater than two-fold induction by menadione. Nitrosative stress also induced the expression of 21 genes to levels above the cut-off value compared to the control (Fig. 3d).

An ANOVA with a *P* value cut-off of <0.01 was performed for each gene to identify those that showed significant variation in their expression levels following oxidative and nitrosative stresses. This analysis revealed that a large majority of genes were significantly expressed in at least one of the oxidative stress conditions compared to nitrosative stress (70 genes, 66.03%). The expression ratios of menadione and SNP treated genes were compared to determine the preferential expression of the genes. Among the 66 genes (62.26%) preferentially expressed following one kind of treatment, 54 genes (50.9%) showed higher expression levels with menadione while 12 genes (11.32%) were upregulated by NO. When all the three stress conditions were compared by ANOVA, a total of 22 genes showed similar expression levels. These genes are involved either in signaling or had an unknown function. The putative signaling genes that were co-expressed included endosomal cargo receptor Erv14 (*Ar79*), C2 domain containing protein (*Ar77*), RAC-alpha serine/threonine-protein kinase (*Ar70*), phosphoinositide 3-phosphate phosphatase (*Ar20*) and protein kinase C domain containing protein (*Ar72*), and the majority exceeded two-fold induction for at least one stress condition.

### qRT-PCR and northern blot analysis validates expression kinetics of several genes

To validate the reliability of the reverse northern analysis results, 12 genes were selected from the library and their expression profiles assessed using real-time quantitative PCR including CAT, GAPDH, Cytochrome C (CYC), F-box and WD domain containing protein (FBO), FMN-dependent dehydrogenase (FMN), neutral trehalase (TRE), SSC1-like heat-shock protein (SSC1), ubiquitin-conjugating enzyme E2N, dnaK-type molecular chaperone BiP (BIP), peptidyl-prolyl *cis*-*trans* isomerase (PPI), mannosylphosphate transferase (MNN4) and NADH-ubiquinone oxidoreductase (NUO). The selection of these genes was based both on their putative function inferred from sequence comparison together with their unique and distinct expression pattern revealed by macroarray analysis. The samples were collected at different time points after menadione treatment and used for qRT-PCR (Fig. S4). The same set of genes was also used to validate their expression in H_2_O_2_ and SNP-treated samples (Fig. S5). Although the data obtained with real-time PCR were consistent with those obtained from the array experiments, the relative expression ratios obtained were often higher. Similar differences between the two methods were reported previously, suggesting that the ratios calculated using macroarray are often underestimated [42]. Real-time PCR allows for the specific amplification of a gene using gene-specific primers, whereas for macroarrays, the possibility of cross hybridization among the homologous genes cannot be ruled out [43]. Furthermore, a few genes were also randomly selected for their expression analysis by northern hybridization (Fig. S6). The genes encoding NADH oxidase (NOX), SOD, GAPDH, histone H1, and a hypothetical protein (GW996392) were selected. The results obtained by northern analysis further substantiated the macroarray data.

### *In planta* expression analysis of selected genes showed high transcript accumulation

**Figure 4:**
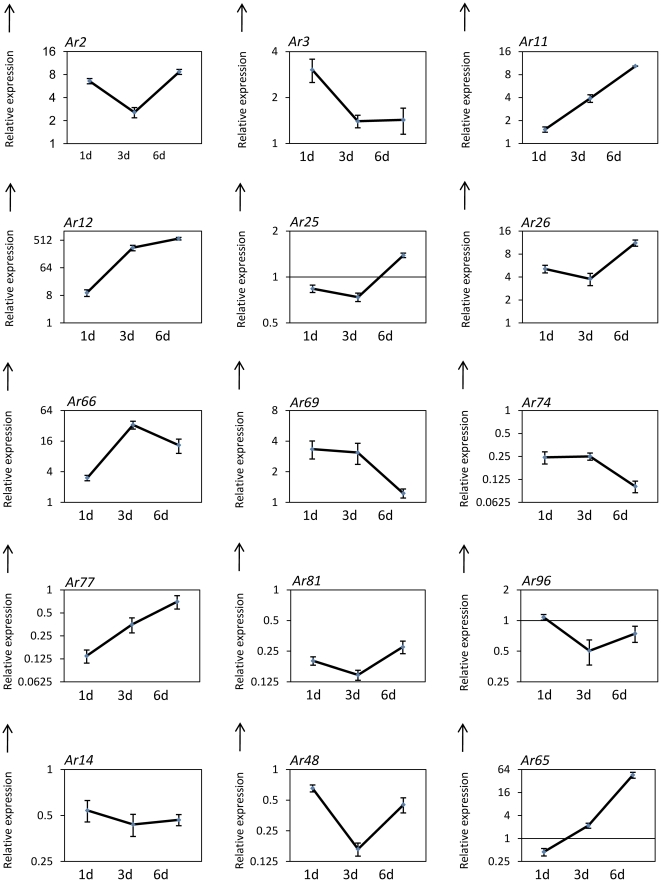
*In planta* quantification of expression of genes belonging to cluster 6. Line diagrams representing the expression pattern of cluster 6 genes from *A. rabiei*-infected chickpea samples (Pusa-362) are shown as fold-change compared to control. Expression was analyzed at 1, 3 and 6 dpi. Error bars represent ± SE. *Ar2*, Hypothetical protein SNOG_16463; *Ar3*, Cytochrome c; *Ar11*, Cartenoid oxygenase; *Ar12*, Acetylglutamate kinase; *Ar25*, Neutral trehalase; *Ar26*, Hypothetical protein; *Ar66,* Hypothetical protein ACLA_073190; *Ar69,* Hypothetical protein SNOG_10250; *Ar74*, Hypothetical protein; *Ar77*, C2 domain containing protein; *Ar81*, Alternative oxidase; *Ar96*, Plasma membrane ATPase; *Ar14*, ATP-citrate synthase; *Ar48,* Hypothetical protein SNOG_00366; *Ar65*, NADH-ubiquinone oxidoreductase.

**Figure 5:**
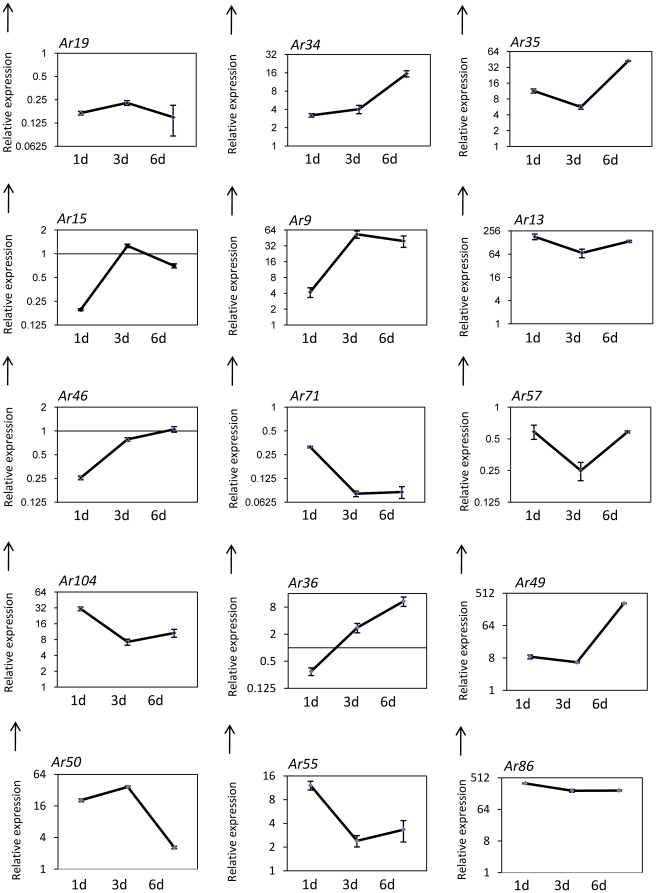
*In planta* expression analysis of stress responsive genes. Line diagrams representing the expression pattern of selected genes from *A. rabiei*-infected chickpea samples (Pusa-362) are shown as fold-change compared to control. Expression was analyzed at 1, 3 and 6 dpi. Error bars represent ±SE. *Ar19*, FMN dependent dehydrogenase; *Ar34*, NADH oxidase; *Ar35*, Catalase; *Ar15*, Thioredoxin; *Ar9,* E3 SUMO-protein ligase PIAS1; *Ar13*, F-box and WD domain containing protein; *Ar46*, Ubiquitin-conjugating enzyme E2 N; *Ar71*, Ubiquitin; *Ar57*, Mannosylphosphate transferase; *Ar104*, Usp domain-containing protein; *Ar36,* C6 transcription factor; *Ar49*, Molecular chaperone BiP; *Ar50,* 41 kDa peptidyl-prolyl cis-trans isomerase; *Ar55*, Ribosomal protein S5; *Ar86*, protein phosphatase PP2A.

To check the relevance of genes isolated in this study to *A. rabiei* infection and disease progression, *in planta* expression analysis of major cluster 6 was carried out by qRT-PCR using the susceptible chickpea cultivar, Pusa 362 and samples collected 1, 3 and 6 d after inoculation (Fig. 4; Table S4). Major cluster 6 consists of some of the hypothetical proteins and genes from the oxidative stress category. In addition, other selected genes from the oxidative stress and stress responsive category together with some genes that showed high expression under oxidative stress were analyzed for *in planta* expression (Fig. 5; Table S4). Two genes of cluster 6, namely *Ar12* and *Ar65* encoding acetylglutamate kinase and NADH-ubiquinone oxidoreductase respectively, showed very high induction but other genes, such as *Ar74*, *Ar77* and *Ar81*, showed repression during infection and disease progression. The majority of genes identified in the stress responsive category showed a greater than 2-fold induction at various time points after infection (*Ar3*, *Ar9*, *Ar13*, *Ar34*, *Ar35* and *Ar104*) while a few others showed repression (*Ar19*, *Ar57* and *Ar71*). Five genes, including *Ar9*, *Ar12*, *A13*, *Ar49* and *Ar86* showed very high induction (>50 fold).

## Discussion

The current study has identified genes that were upregulated by menadione-treatment, and illustrates the processes affected by oxidative stress in phytopathogenic fungi. Since isolation of *Ascochyta* genes from the infected chickpea plants precisely at the time of the initial oxidative burst was not realistic, we used liquid cultures with mild menadione treatment to mimic *in planta* oxidative stress. Many genes showed high induction immediately after menadione treatment. The utility of the SSH strategy is highlighted by the fact that a large number of genes identified from the library were not previously characterized or were categorized as genes having unknown function (31%). The short length of some clones and the long 3′-untranslated region in the truncated cDNAs could also account for the high number of sequences with unknown function [36], [41], although the sequence length did exceed 500 bp in a few cases (*Ar106* and *Ar113*). A large number of ‘no hits’ (10.93%) found in the library also demonstrated that subtractive libraries can be used to identify new genes that are overlooked by large scale EST projects. Furthermore, with the 70 up-regulated genes representing 54.69% of the 128 SSH clones, the subtractive approach was shown to be highly efficient. Since the subtractive library was generated 1 h after menadione treatment of the fungus, it is possible that some genes may be induced at later time points and would have escaped detection.

**Figure 6:**
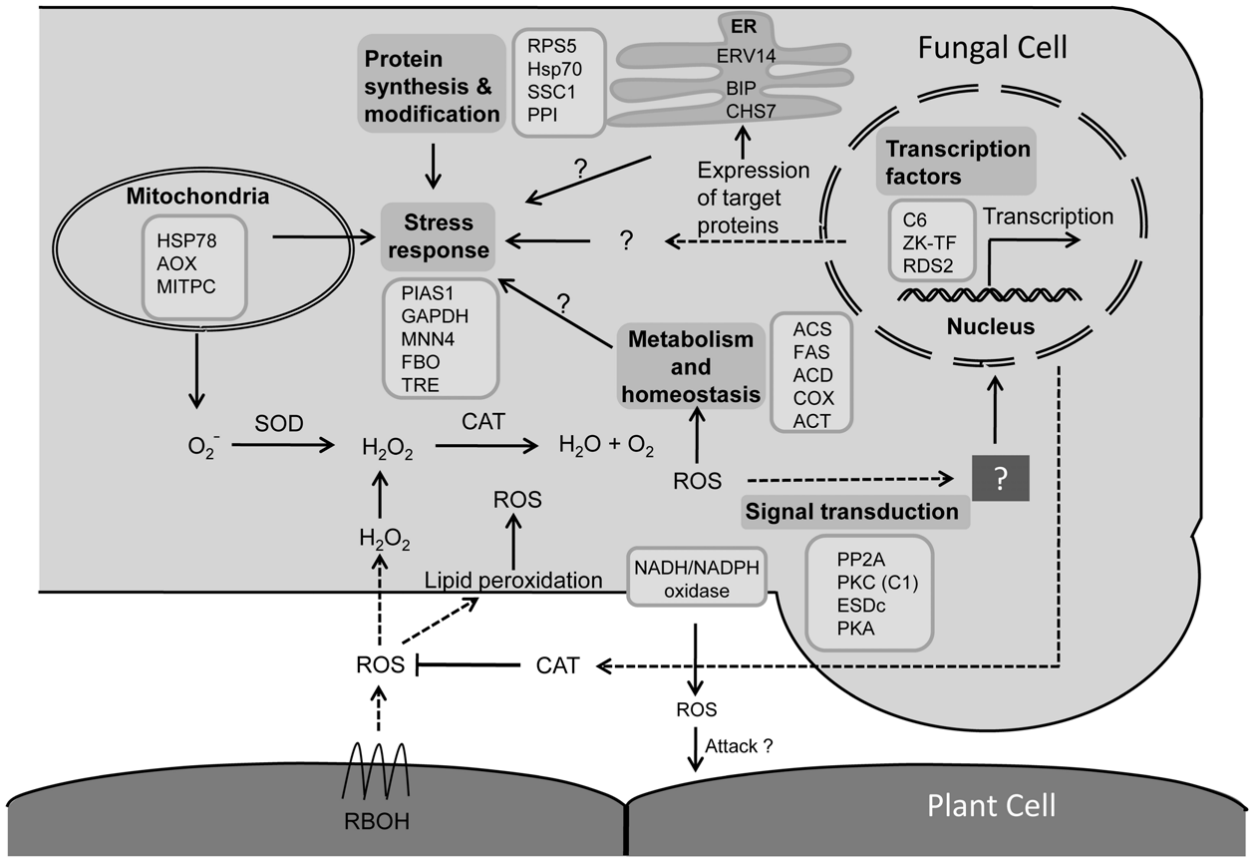
Schematic representation of the putative regulatory and functional networks induced during oxidative stress in *Ascochyta rabiei*. This figure summarizes data obtained in the present work and hypothesized mechanisms. Genes are grouped according to their most probable localization and function in the cell. Abbreviation for genes: PP2A, Protein phosphatase PP2A; PKC (C1), Protein kinase C (C1); EsdC, GTP-binding protein EsdC; PKA, RAC-alpha serine/threonine-protein kinase; C6, C6 transcription factor; ZK-TF, Zinc knuckle transcription factor; Rds2, Zn cluster transcription factor Rds2; HSP78, Heat shock protein 78; AOX, Alternative oxidase; MITPC, Mitochondrial phosphate carrier protein; SOD, Superoxide dismutase; CAT, Catalase; ERV14, Endosomal cargo receptor Erv14; BIP, Molecular chaperone BIP; CHS7, Export control protein chitin synthase 7; ACS, ATP-citrate synthase; FAS, Fatty acid synthase; ACD, Acyl-CoA desaturase; COX, Carotenoid oxygenase; ACT, Acetylglutamate kinase; PIAS, E3 SUMO-protein ligase PIAS1; GAPDH, Glyceraldehyde 3-phosphate dehydrogenase; Mnn4, Mannosylphosphate transferase; FBO, F-box and WD domain containing protein; TRE, Trehalase; RPS5, Ribosomal protein S5; HSP 70, Heat shock protein 70; SSC1, SSC1-like heat-shock protein; PPI, peptidyl-prolyl *cis*-*trans* isomerase; RBOH, Respiratory burst oxidase homolog.

Analyses of macroarray results suggest that menadione treatment induces many genes potentially involved in protecting cells from oxidative stress and ROS detoxification. Among these SOD, CAT and TRX (thioredoxin) are known to be involved directly in ROS detoxification. Thioredoxins are small, thermostable proteins identified as donors of hydrogen to ribonucleotide reductase [44]. The *Cryptococcus neoformans* thioredoxin was reported to regulate oxidative and nitrosative stress [45]. The upregulation of *A. rabiei* thioredoxin by menadione, SNP and H_2_O_2_ treatments suggests it may play a similar role. Additionally, the up-regulation of many genes involved in lipid metabolism, protein synthesis, protein folding and modification is suggestive of their involvement in fungus survival during the HR. For example, the role of heat-shock proteins as stress regulators during oxidative stress is reflected by the up-regulation of HSP70 (HscA), PPI and 30 kDa HSP. All of these chaperones have well-established roles during different stresses in other organisms [46], [47], and therefore the isolation and up-regulation observed here also suggests their importance in phytopathogenic fungi. A schematic model based on our work that illustrates the regulatory and functional networks activated under oxidative stress is depicted in Figure 6.

Out of the four major clusters identified by SOTA clustering analysis (Fig. 3), we selected cluster 6 for *in planta* analysis since this cluster included many genes of unknown function together with a few genes that participate in the oxidative stress response. The majority of the genes grouped in cluster 6 showed induction under oxidative stress. *In planta* expression analysis was carried out using infected samples collected 1, 3, and 6 dpi. Previous *A. rabiei* studies have revealed that the initiation of spore germination, germ tube elongation and penetration occurred around 1 dpi, followed by colonization after 2–3 dpi [30], [31]. Visual symptoms of the disease were observed after 5–6 dpi. Expression analysis of cluster 6 revealed a set of genes having high induction while some showed repression. Although genes of this cluster were induced during oxidative stress, many of them, including thioredoxin (*Ar15*) and alternative oxidase (*Ar81*), showed repression during infection. Interestingly, the majority of genes involved in stress responses were induced during infection with a few exceptions. A few genes, including superoxide dismutase (*Ar94*), glyceraldehyde 3-phosphate dehydrogenase (*Ar61*) and Niemann-Pick C1 protein precursor (*Ar62*) showed prominant multiple peaks in qRT-PCR and were not considered for *in planta* expression analysis..

Among the isolated genes, those encoding acetylglutamate kinase, E3 SUMO-protein ligase PIAS1, the molecular chaperone BiP, F-box and WD protein (FBO), and protein phosphatase 2A (PP2A) showed very high induction. F-box domain containing proteins are considered to be scavenger proteins in the cell [48] and are reported to have important roles in various phytopathogenic fungi, including *M. oryzae* and *Fusarium oxysporum* [49]. PP2A is a critical regulator of many cellular activities [50], [51], and in phytopathogenic fungi is necessary for vitality and pathogenicity as in the biotroph *U. maydis* [52] and the necrotroph *S. sclerotiorum* [53]. The very high induction of PP2A (>200-fold) observed in infected chickpea samples predicts its importance for *A. rabiei* during infection. The acetylglutamate kinase of the crucifer anthracnose fungus *Colletotrichum higginsianum* has been reported to be a pathogenicity factor [54]. The gene encoding neutral trehalase (Ar25) showed no significant change in expression during infection, which supports the finding that the *M. oryzae* trehalase, *TRE1* did not play a significant role in pathogenesis [55].

Previously, the expression patterns of many genes in response to oxidative stress were studied for the filamentous fungi *Aspergillus nidulans* and *M. oryzae* using microarray analysis [27], [40]. Comparison of the results for certain *A. nidulans* genes with those in the present study yielded varying results with both studies showing menadione dependent expression of alternative oxidase (AOX), while MnSOD had no significant expression in *A. nidulans*. The difference between the two studies may be due to the very high concentration of ROS generators used in Pócsi et al. [40] to provide a ‘minimum lethal dose’.

When comparing different treatment time points by macroarray analysis, a large number of genes isolated from the library were found to be induced immediately after the 0.5 h of menadione-treatment. These genes primarily coded for stress-responsive genes together with a few genes from other categories. Furthermore, many genes of the library were upregulated by RNS along with ROS treatment. These genes are likely part of the cell machinery that acts in co-ordination to regulate responses to stress. ANOVA analyses suggest that signaling between oxidative and nitrosative stress overlaps in *A. rabiei* since many signaling genes were found to be co-expressed. Therefore, our results support the theory that the signal transduction pathways initiated by these two types of stress consistently overlap [56]. Overall, this study suggests that fungi employ various pathways and regulatory networks of genes to resist various types of stress imparted by ROS and RNS.

In conclusion, we report here the detailed investigation of gene expression under oxidative and nitrosative stress conditions in the phytopathogenic fungi, *Ascochyta rabiei*. The high expression of many genes is likely indicative of their role during infection, although further research using gene knock-out and infection studies on chickpea plants will provide additional evidence that some of these identified genes are required for coping with host-induced oxidative stress and cell death. The data presented here also provide a portfolio of candidate genes for further studies on their role in oxidative and nitrosative stress tolerance.

## Supporting Information

Figure S1 **DAB staining to detect ROS production during** ***Ascochyta*** **-chickpea interaction.** (I) A light micrograph of an *A. rabiei* germinating spore infecting chickpea tissue (II) merged micrograph of DsRed-expressing *A. rabiei* infecting chickpea stem peel. DAB precipitates are visible as black precipitates in the vicinity of the spores (I) or hypha (II). Bars = 10 µm.
(TIF)

Figure S2 **Measurement of fungal biomass (dry weight) and mycelia ball size.** The measurement of fungal biomass (dry weight) and mycelia ball size carried out after incubation for 24 h with different menadione concentrations.
(TIF)

Figure S3 **RNA gel-blot analysis of actin and** ***β*** **-tubulin.**
(TIF)

Figure S4 **Real-time quantitative PCR validation of macroarray analysis after menadione- treatment for different time intervals.** Bar diagrams representing the expression pattern of 12 genes are shown as the fold-change compared to their controls. The solid black bars represent the qRT PCR results. Error bars represent ±SE.
(TIF)

Figure S5 **Validation of macroarrays by real-time quantitative PCR after menadione, H_2_O_2_ and NO treatments.** Bar diagrams representing the expression pattern of twelve genes are shown as the fold-change compared with their controls. The solid black bars represent the qRT PCR results. Error bars represent ±SE.
(TIF)

Figure S6 **RNA gel-blot analysis of selected genes from the library.**
(TIF)

Table S1 **Genes differentially expressed in response to menadione, H_2_O_2_ and NO.**
(DOC)

Table S2 **List of primers for real-time quantitative PCR.**
(DOC)

Table S3 **Group of clusters after SOTA analysis.**
(DOC)

Table S4 **Relative expression of selected genes** ***in planta*** **determined by qRT-PCR.**
(DOC)
