# Supplementary figures and images for: Comparative Transcriptome Analysis of the Necrotrophic Fungus Ascochyta rabiei during Oxidative Stress: Insight for Fungal Survival in the Host Plant
Source: PLoS One. 2012 Mar 12;7(3):e33128. doi: 10.1371/journal.pone.0033128 (PMC3299738; doi:10.1371/journal.pone.0033128)

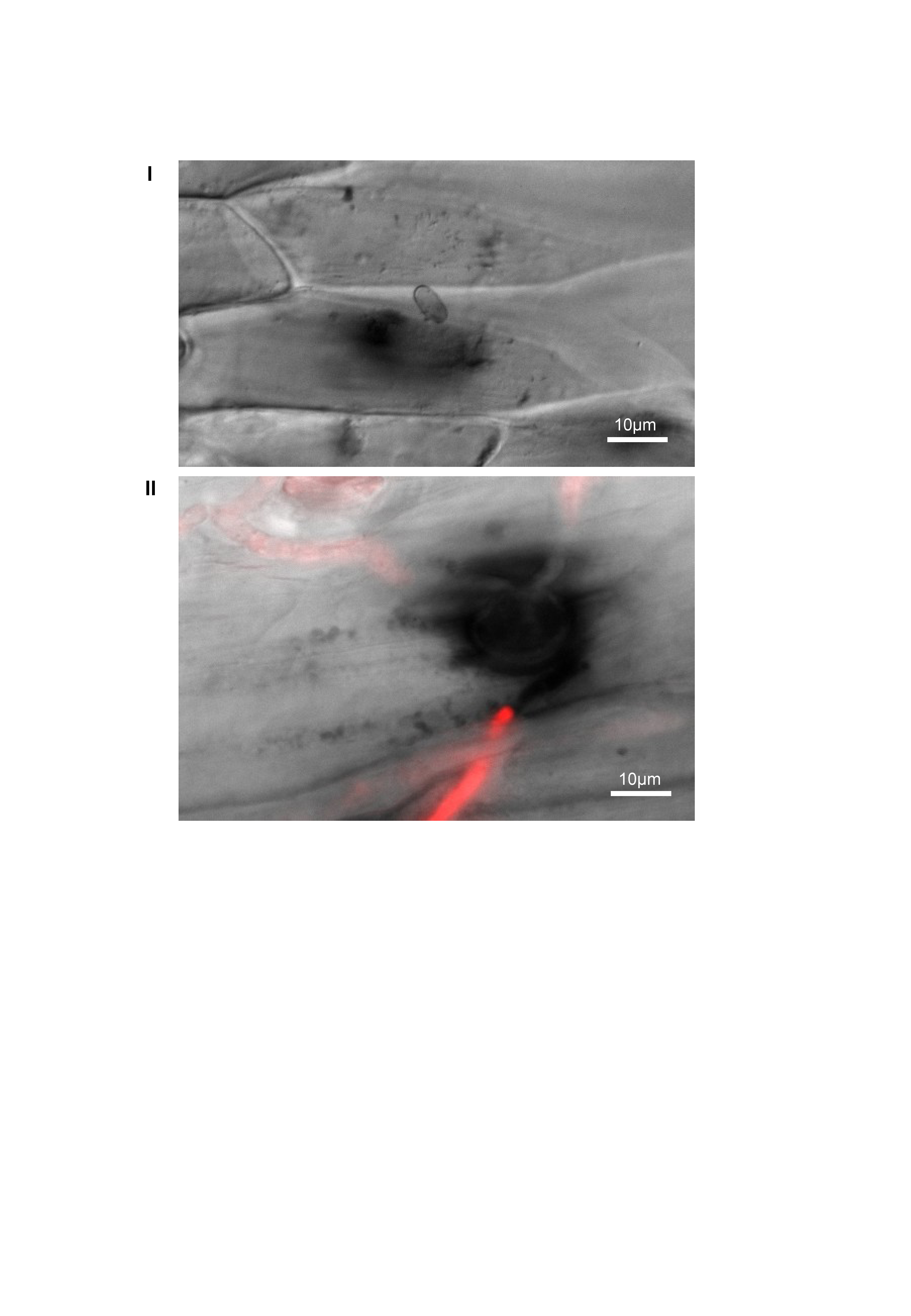

Supplement: Figure S1 — DAB staining to detect ROS production during Ascochyta -chickpea interaction. (I) A light micrograph of an A. rabiei germinating spore infecting chickpea tissue (II) merged micrograph of DsRed-expressing A. rabiei infecting chickpea stem peel. DAB precipitates are visible as black precipitates in the vicinity of the spores (I) or hypha (II). Bars = 10 µm. (TIF) [file pone.0033128.s001.tif]

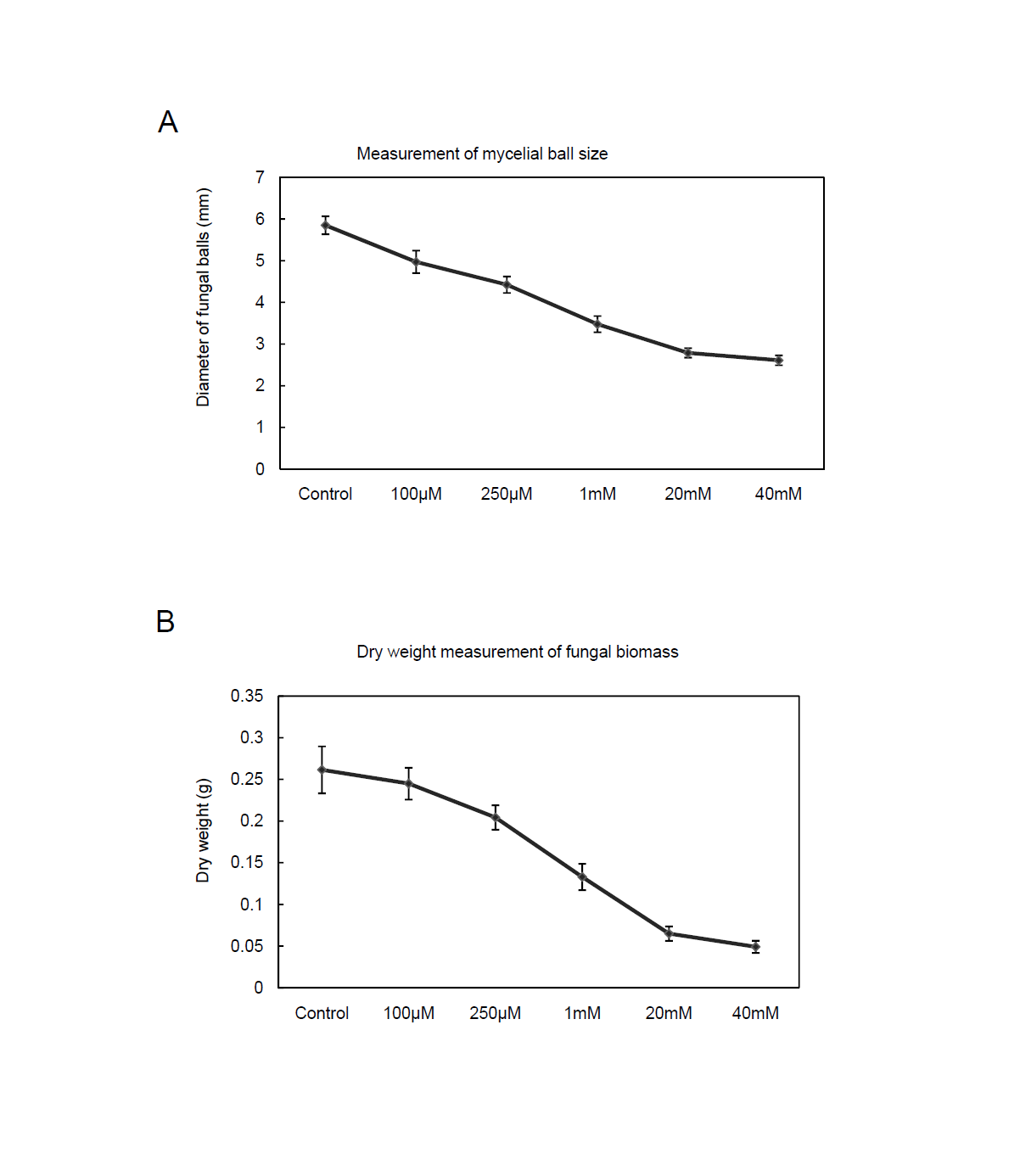

Supplement: Figure S2 — Measurement of fungal biomass (dry weight) and mycelia ball size. The measurement of fungal biomass (dry weight) and mycelia ball size carried out after incubation for 24 h with different menadione concentrations. (TIF) [file pone.0033128.s002.tif]

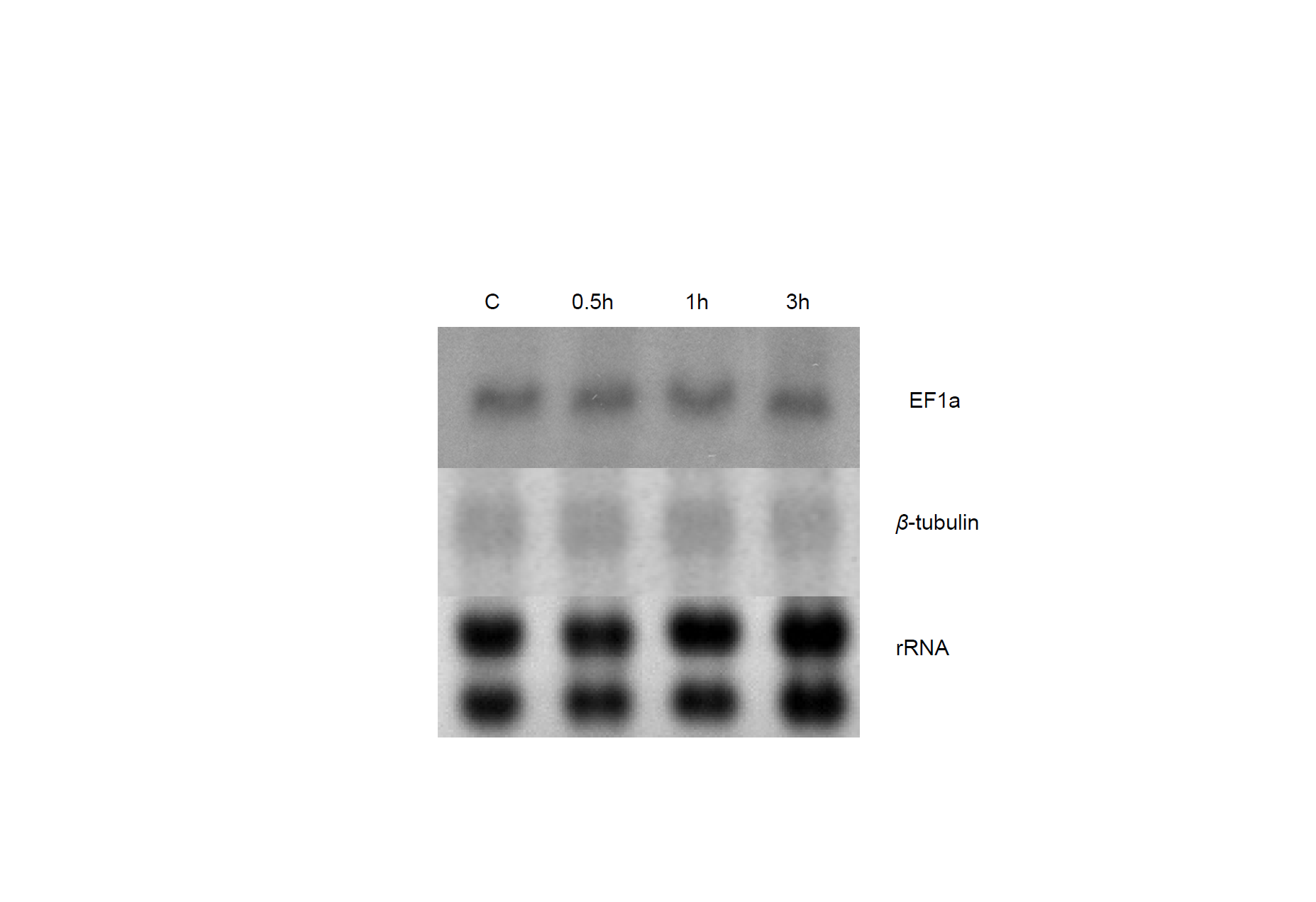

Supplement: Figure S3 — RNA gel-blot analysis of actin and β -tubulin. (TIF) [file pone.0033128.s003.tif]

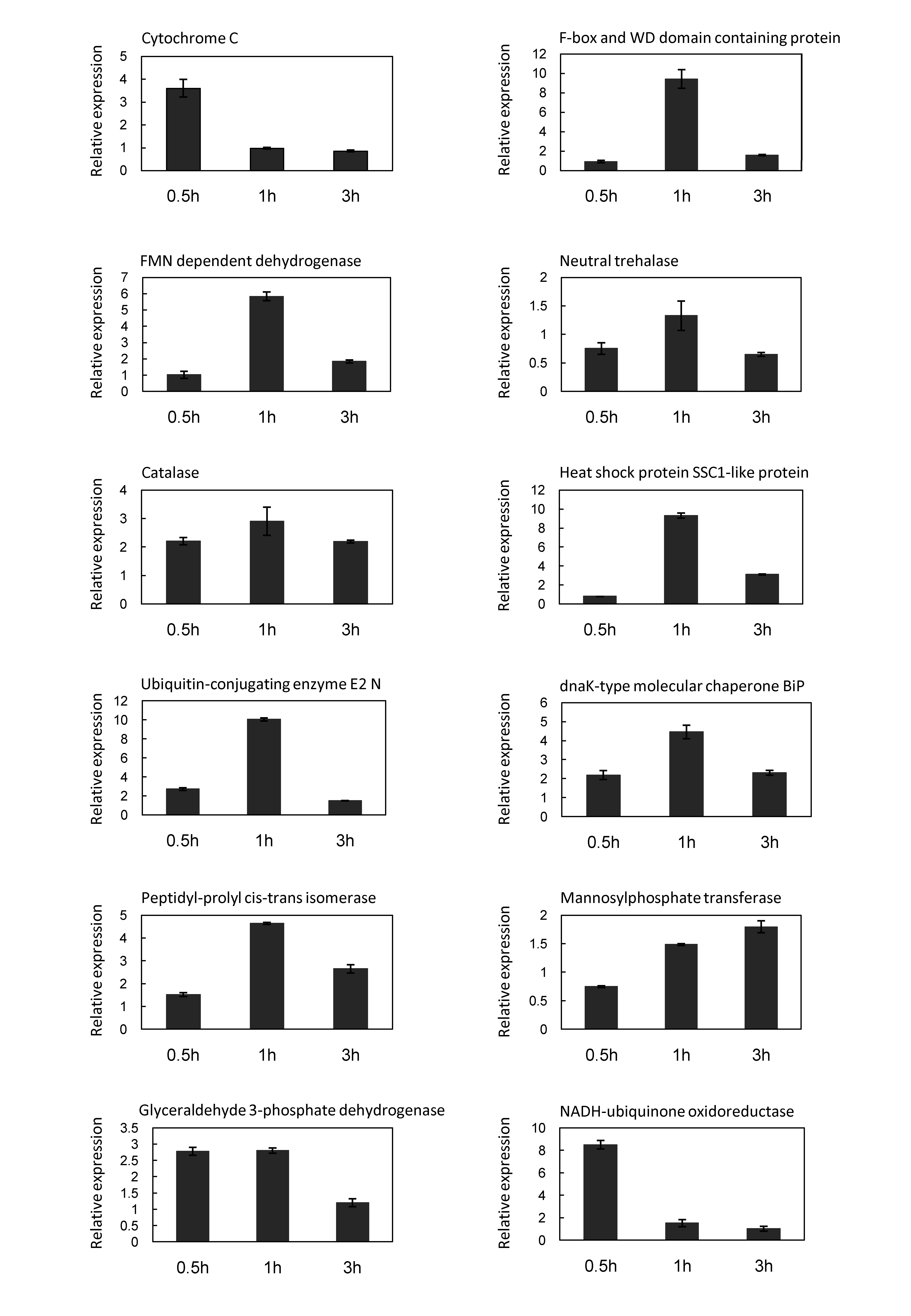

Supplement: Figure S4 — Real-time quantitative PCR validation of macroarray analysis after menadione- treatment for different time intervals. Bar diagrams representing the expression pattern of 12 genes are shown as the fold-change compared to their controls. The solid black bars represent the qRT PCR results. Error bars represent ±SE. (TIF) [file pone.0033128.s004.tif]

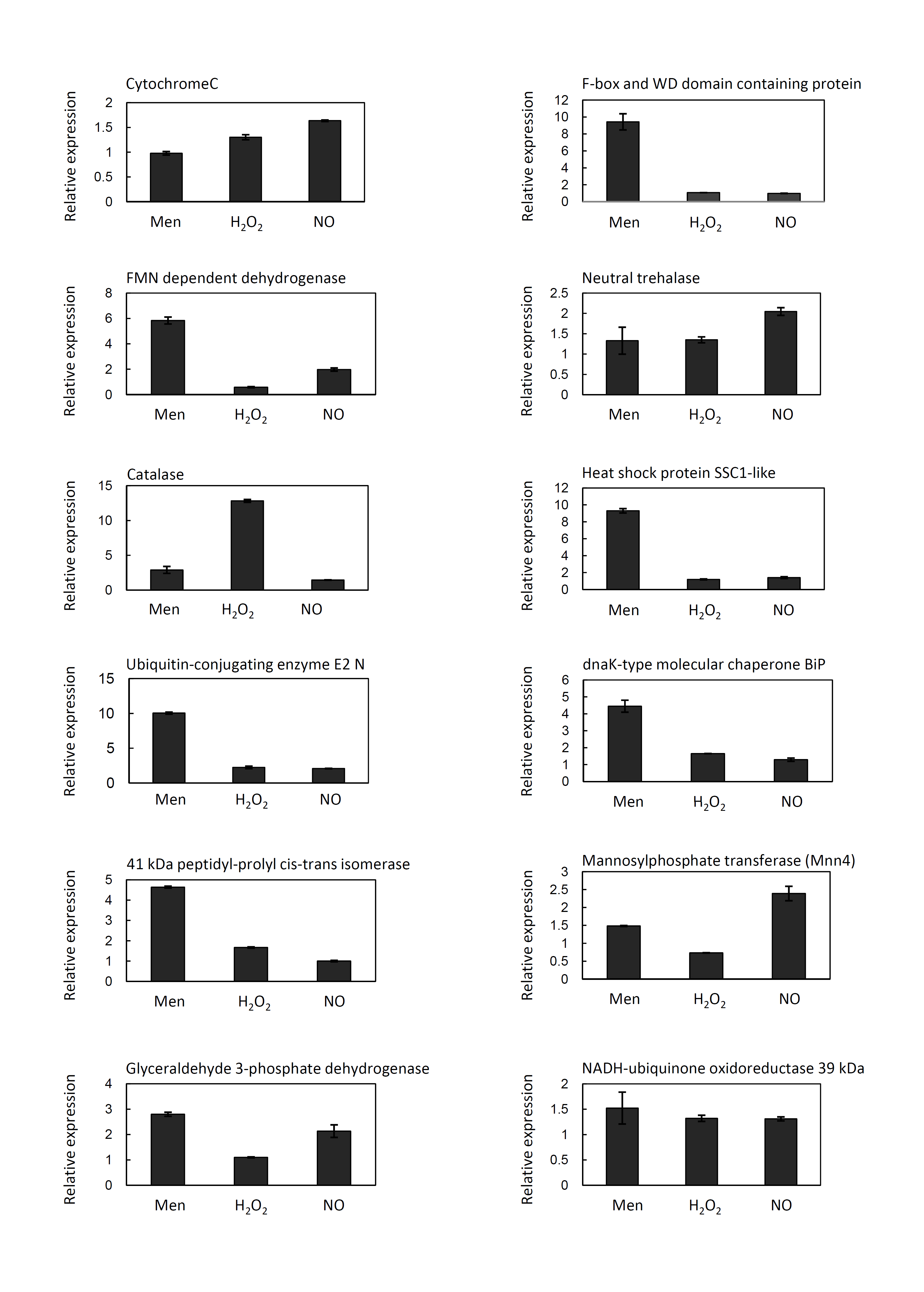

Supplement: Figure S5 — Validation of macroarrays by real-time quantitative PCR after menadione, H2O2 and NO treatments. Bar diagrams representing the expression pattern of twelve genes are shown as the fold-change compared with their controls. The solid black bars represent the qRT PCR results. Error bars represent ±SE. (TIF) [file pone.0033128.s005.tif]

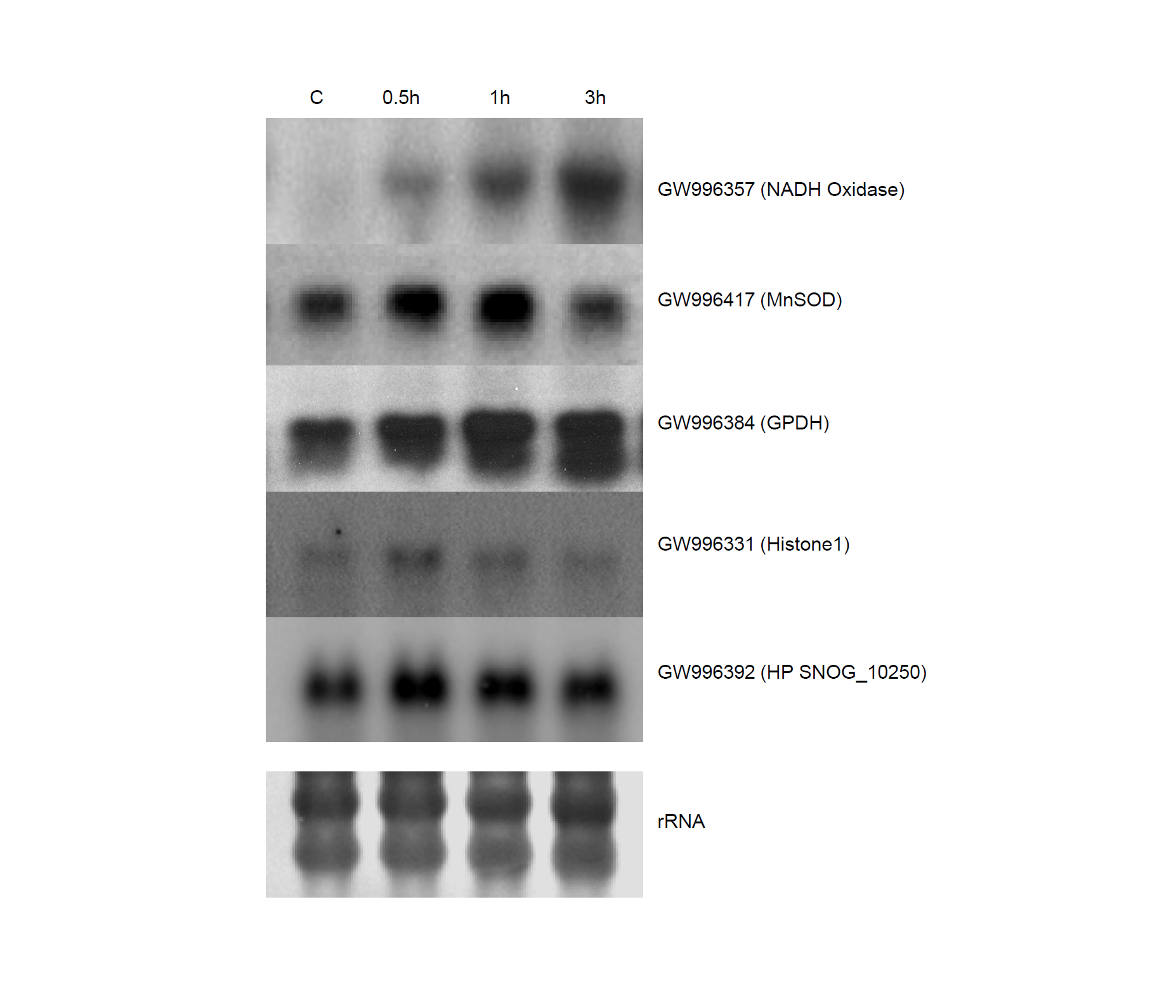

Supplement: Figure S6 — RNA gel-blot analysis of selected genes from the library. (TIF) [file pone.0033128.s006.tif]
