# Supplementary material for: Comparative Transcriptome Analysis of the Necrotrophic Fungus Ascochyta rabiei during Oxidative Stress: Insight for Fungal Survival in the Host Plant
Source: PLoS One. 2012 Mar 12;7(3):e33128. doi: 10.1371/journal.pone.0033128 (PMC3299738; doi:10.1371/journal.pone.0033128)
Supplement: Table S1 — Genes differentially expressed in response to menadione, H2O2 and NO. (DOC) [file pone.0033128.s007.doc]

**Table S1: Genes differentially expressed in response to Menadione, H_2_O_2_ and NO.**

| **Clone_id**^a^ | **Genes**^b^ | **Accession**^c^ | **Menadione**^d,e,f^  **0.5 h 1 h 3 h** | | | **H_2_O_2_** | **NO** |
| --- | --- | --- | --- | --- | --- | --- | --- |
| **Oxidative stress responsive** | | | | | | | |
| Ar3 | Cytochrome c | GW996326 | **2.19±0.36** | 1.30±0.13 | 1.05±0.18 | 1.38±0.31 | 1.65±0.24 |
| Ar19 | FMN dependent dehydrogenase | GW996342 | 1.02±0.22 | **2.90±0.40** | 1.63±0.30 | 0.93±0.38 | 1.71±0.16 |
| Ar34 | NADH oxidase | GW996357 | 0.97±0.30 | **2.23±0.34** | **6.48±0.67** | 1.01±0.17 | 1.32±0.04 |
| Ar35 | Catalase | GW996358 | 1.68±0.28 | **3.72±0.42** | **3.05±0.51** | **5.11±0.42** | 1.53±0.06 |
| Ar62 | Niemann-Pick C1 protein precursor | GW996385 | **2.05±0.21** | **2.72±0.68** | 0.78±0.21 | 1.34±0.22 | 0.34±0.20 |
| Ar65 | Putative, NADH-ubiquinone oxidoreductase 39 kDa subunit | GW996388 | **4.39±0.70** | 1.25±0.37 | 1.09±0.33 | 1.59±0.14 | 0.95±0.18 |
| Ar81 | Alternative oxidase, mitochondrial precursor | GW996404 | 1.66±0.38 | **2.10±0.31** | 1.88±0.33 | 1.72±0.73 | **2.08±0.46** |
| Ar94 | Superoxide dismutase | GW996417 | 1.73±0.23 | **2.06±0.12** | 1.07±0.10 | 1.46±0.27 | 1.30±0.13 |
| Ar15 | TRX_family (Thioredoxin), putative | GW996338 | **2.03±0.69** | 1.50±0.39 | 0.90±0.16 | **3.92±0.26** | **2.34±0.17** |
| **Protein transport regulation** | | | | | | | |
| Ar1 | Export control protein CHS7-like, putative | GW996324 | 0.88±0.08 | **1.99±0.37** | 1.18±0.12 | 1.04±0.16 | 0.61±0.14 |
| Ar44 | Acriflavin resistance protein | GW996367 | 0.77±0.05 | 0.93±0.31 | 0.55±0.03 | 0.45±0.10 | 1.26±0.11 |
| Ar84 | Multidrug resistant protein | GW996407 | 1.47±0.28 | 1.02±0.31 | 1.39±0.30 | **2.94±0.84** | 1.57±0.57 |
| Ar27 | SecE/Sec61 subunit containing protein, Putative | GW996350 | 1.44±0.16 | 1.59±0.31 | **2.00±0.30** | 1.07±0.30 | 1.00±0.10 |
| **Cell signaling** | | | | | | | |
| Ar16 | Phosphoserine phosphatase, putative | GW996339 | 1.36±0.23 | 1.65±0.28 | **2.05**±0.30 | 1.10±0.54 | 0.86±0.22 |
| Ar20 | Phosphoinositide 3-phosphate phosphatase | GW996343 | 1.22±0.28 | 0.93±0.24 | 0.74±0.54 | 0.80±0.40 | 0.99±0.08 |
| Ar70 | RAC-alpha serine/threonine-protein kinase | GW996393 | **2.07±0.98** | 1.26±0.32 | 0.81±0.27 | 0.93±0.34 | 0.76±0.18 |
| Ar79 | Endosomal cargo receptor Erv14 | GW996402 | **2.62±0.43** | **2.48±0.61** | 1.04±0.47 | 1.76±0.69 | **2.33±0.78** |
| Ar85 | GTP-binding protein EsdC | GW996408 | **15.13±1.58** | **3.37±0.40** | **2.59±0.60** | **4.03±1.04** | **6.02±1.10** |
| Ar86 | Serine/threonine-protein phosphatase PP2A catalytic subunit | GW996409 | 1.37±0.12 | **2.53±0.34** | 0.98±0.28 | 1.04±0.25 | 0.79±0.42 |
| Ar90 | Annexin A7 | GW996413 | 0.85±0.40 | 1.83±0.71 | **2.27±0.25** | 0.84±0.09 | 0.93±0.32 |
| Ar72 | Protein kinase C (C1) domain contain protein, Putative | GW996395 | **4.28±0.53** | **2.93±0.67** | 1.13±0.51 | **2.09±0.43** | **2.02±0.77** |
| Ar77 | C2 domain containing protein | GW996400 | 1.14±0.17 | 1.23±0.57 | 1.03±0.65 | **2.08±0.97** | 1.30±0.54 |
| **Protein synthesis** | | | | | | | |
| Ar4 | 60S ribosomal protein P0 | GW996327 | 1.00±0.38 | **4.23±0.81** | 1.66±0.49 | 0.92±0.15 | 0.40±0.13 |
| Ar39 | Ribosomal protein L26 | GW996362 | 1.01±0.06 | **5.86±0.39** | **2.05±0.21** | 1.00±0.12 | 1.39±0.01 |
| Ar40 | 60S acidic ribosomal protein P0 | GW996363 | 1.31±0.21 | **3.27±0.26** | 1.86±0.24 | 0.91±0.10 | 1.65±0.06 |
| Ar55 | Ribosomal protein S5 | GW996378 | **6.20±1.64** | 1.08±0.27 | 0.94±0.20 | **3.50±0.27** | **4.03±0.47** |
| Ar68 | 60S ribosomal protein L36 | GW996391 | **2.25±0.77** | **2.62±0.58** | 0.93±0.50 | 1.72±0.30 | 0.57±0.22 |
| Ar112 | large subunit ribosomal RNA gene | GW996435 | 1.23±0.16 | 1.02±0.66 | 1.62±0.54 | 1.29±0.27 | 0.74±0.08 |
| **Metabolism and homeostasis** | | | | | | | |
| Ar6 | 3-hydroxyisobutyryl-CoA hydrolase, mitochondrial precursor | GW996329 | 1.04±0.32 | **3.74±0.55** | 1.28±0.33 | 1.03±0.45 | 0.50±0.14 |
| Ar7 | Uracil phosphoribosyltransferase | GW996330 | 1.46±0.34 | 1.02±0.35 | 1.08±0.20 | 1.33±0.33 | 0.60±0.31 |
| Ar11 | Cartenoid oxygenase, putative | GW996334 | 1.19±0.41 | 0.96±0.34 | 0.70±0.57 | **2.49±0.07** | 1.03±0.29 |
| Ar12 | Acetylglutamate kinase, putative | GW996335 | 1.08±0.32 | 1.47±0.58 | 0.99±0.36 | **2.50±0.88** | 0.93±0.48 |
| Ar14 | ATP-citrate synthase | GW996337 | 1.03±0.31 | 1.25±0.36 | 1.03±0.15 | **3.28±0.79** | 0.60±0.18 |
| Ar28 | Fatty acid synthase subunit beta dehydratase | GW996351 | 1.14±0.15 | **2.01±0.36** | 1.14±0.36 | 0.70±0.30 | 0.88±0.10 |
| Ar37 | Acyl-CoA desaturase | GW996360 | 0.93±0.16 | **2.46±0.45** | 1.48±0.17 | 0.78±0.13 | 0.50±0.08 |
| Ar95 | Myo-inositol-phosphate synthase, putative | GW996418 | 1.56±0.38 | 1.37±0.01 | 1.34±0.17 | **2.00±0.22** | 1.01±0.30 |
| Ar103 | Fumarylacetoacetate hydrolase | GW996426 | 0.98±0.44 | **2.55±0.59** | **2.35±0.48** | 0.59±0.22 | 0.35±0.22 |
| **Protein modification** | | | | | | | |
| Ar38 | Heat shock protein SSC1-like protein | GW996361 | 1.09±0.03 | **4.85±0.46** | **2.07±0.20** | 0.98±0.06 | 1.67±0.01 |
| Ar49 | Molecular chaperone BiP | GW996372 | 1.54±0.08 | **3.93±0.62** | 1.91±0.22 | 1.51±0.13 | 0.97±0.20 |
| Ar50 | 41 kDa peptidyl-prolyl cis-trans isomerase | GW996373 | 1.34±0.26 | **5.39±0.88** | 1.87±0.42 | 1.57±0.25 | 1.36±0.33 |
| Ar56 | Hsp70 chaperone (HscA), putative | GW996379 | 1.07±0.01 | **2.07**±0.32 | 0.92±0.22 | 0.74±0.07 | 1.31±0.14 |
| Ar59 | 30 kDa heat shock protein | GW996382 | **4.99±0.79** | **2.55±0.67** | 1.46±0.45 | **3.47±0.88** | **3.62±0.78** |
| Ar63 | Heat shock protein 78, mitochondrial precursor | GW996386 | 0.93±0.45 | 1.62±0.45 | 0.84±0.16 | 1.24±0.65 | 0.89±0.35 |
| **Stress responsive** | | | | | | | |
| Ar9 | E3 SUMO-protein ligase PIAS1 | GW996332 | 1.00±0.45 | **2.03±0.52** | 0.75±0.28 | 1.88±0.70 | 0.85±0.30 |
| Ar13 | F-box and WD domain containing protein | GW996336 | 0.97±0.10 | **3.02±0.75** | 1.08±0.16 | 1.29±0.29 | 1.22±0.12 |
| Ar25 | Neutral trehalase | GW996348 | 0.79±0.05 | 1.06±0.22 | 0.79±0.17 | 1.22±0.16 | 1.64±0.28 |
| Ar46 | Ubiquitin-conjugating enzyme E2 N | GW996369 | 1.90±0.35 | **6.19±0.96** | 1.42±1.37 | **2.02±0.40** | **2.58±0.91** |
| Ar71 | Ubiquitin | GW996394 | **7.01±0.68** | **4.84±0.42** | **2.42±0.61** | **3.81±0.88** | **6.12±0.81** |
| Ar104 | Usp domain-containing protein | GW996427 | 0.84±0.05 | 1.29±0.06 | 0.79±0.08 | 0.92±0.05 | 0.33±0.03 |
| Ar61 | Glyceraldehyde 3-phosphate dehydrogenase | GW996384 | **2.96±0.32** | **3.50±0.63** | 1.49±0.53 | 1.39±0.15 | **3.71±0.55** |
| Ar57 | Mannosylphosphate transferase (Mnn4), putative | GW996380 | 1.13±0.08 | 1.88±0.68 | **2.27±1.33** | 0.75±0.02 | **2.05±0.10** |
| **Transcription factor** | | | | | | | |
| Ar31 | Zinc knuckle transcription factor/splicing factor MSL5/ZFM1 | GW996354 | 0.92±0.24 | **2.03±0.40** | 0.80±0.21 | 0.64±0.03 | 1.01±0.27 |
| Ar33 | Zn cluster transcription factor Rds2, putative | GW996356 | 0.96±0.11 | 1.15±0.80 | **2.08±0.40** | 0.85±0.10 | 1.42±0.32 |
| Ar36 | C6 transcription factor, putative | GW996359 | 1.55±0.16 | **2.01±0.45** | **3.22±0.46** | 0.87±0.06 | 1.36±0.20 |
| **Miscellaneous** | | | | | | | |
| Ar8 | Histone H1 | GW996331 | 0.86±0.22 | 1.39±0.39 | 1.03±0.22 | 1.59±0.33 | 0.72±0.48 |
| Ar17 | Cell surface protein, putative | GW996340 | 1.09±0.54 | **6.28±0.78** | 1.60±0.74 | **2.08±0.35** | 1.80±0.05 |
| Ar24 | Ribonucleoside-diphosphate reductase subunit M2 | GW996347 | 1.23±0.49 | 1.37±0.31 | 0.84±0.38 | 1.18±0.46 | **2.22±0.98** |
| Ar42 | Pre-rRNA-processing protein TSR2 | GW996365 | 0.91±0.34 | **2.67±0.83** | 0.66±0.18 | 0.71±0.28 | 0.83±0.07 |
| Ar43 | PhiA protein | GW996366 | 1.29±0.12 | **2.92±0.45** | 0.97±0.38 | **3.88±0.80** | **4.72±0.60** |
| Ar58 | Mitochondria fission 1 protein | GW996381 | 0.95±0.07 | **3.08±0.57** | 1.05±0.45 | 0.71±0.03 | 0.90±0.10 |
| Ar60 | RNA polymerase II subunit | GW996383 | 0.92±0.30 | 1.92±0.59 | 0.86±0.35 | 0.83±0.16 | 0.71±0.16 |
| Ar73 | RING-8 protein | GW996396 | **4.38±0.99** | 1.88±0.44 | 0.86±0.23 | **2.87±0.78** | 1.77±0.86 |
| Ar76 | Mitochondrial phosphate carrier protein | GW996399 | **2.61±0.99** | **2.42±0.58** | 0.83±0.61 | 1.09±0.49 | 1.05±0.64 |
| Ar80 | Cell lysis protein-like | GW996403 | 1.67±0.71 | **2.89±0.35** | 1.03±0.60 | 1.78±0.85 | 1.09±0.38 |
| Ar83 | Extracellular cell wall glucanase Crf1/allergen Asp F9 | GW996406 | 1.41±0.24 | **2.62±0.86** | 0.77±0.55 | 0.73±0.18 | 0.65±0.14 |
| Ar96 | Plasma membrane ATPase | GW996419 | **2.89±0.21** | 1.79±0.15 | **3.03±0.79** | **2.92±0.17** | 1.17±0.04 |
| Ar102 | Molybdopterin binding domain protein | GW996425 | 1.06±0.24 | 1.07±0.02 | 0.69±0.06 | 1.01±0.16 | 0.60±0.19 |
| Ar23 | ChtBD1, Chitin binding domain containing protein, putative | GW996346 | 0.83±0.11 | 0.54±0.10 | 0.54±0.30 | 0.57±0.31 | 0.48±0.07 |
| Ar47 | Uterine-derived 14 kDa protein | GW996370 | 1.23±0.29 | **7.92±0.93** | **2.21±0.74** | 0.91±0.28 | **2.39±1.02** |
| Ar75 | MARVEL, Membrane-associating domain | GW996398 | **3.07±0.86** | **2.45±0.47** | 0.82±0.13 | 1.98±0.79 | **2.02±0.92** |
| Ar119 | Nucleosome assembly protein | GW996442 | 1.16±0.04 | 0.86±0.09 | 0.77±0.02 | 1.03±0.08 | 0.76±0.01 |
| **Protein of unknown function/Hypothetical** | | | | | | | |
| Ar2 | Hypothetical protein SNOG_16463 | GW996325 | 1.12±0.38 | 1.97±0.64 | 1.23±0.36 | 1.89±0.67 | 1.12±0.82 |
| Ar5 | Hypothetical protein SNOG_15646 | GW996328 | 1.16±0.18 | 0.97±0.20 | 0.65±0.56 | 1.03±0.24 | 0.44±0.24 |
| Ar10 | Hypothetical protein PTRG_10228 | GW996333 | 0.77±0.08 | **2.62±0.48** | 1.54±0.32 | 1.45±0.16 | **2.42±0.37** |
| Ar21 | Hypothetical protein SNOG_10728 | GW996344 | 1.04±0.58 | **2.43±0.33** | 1.24±0.17 | 1.28±0.27 | 0.79±0.32 |
| Ar22 | Hypothetical protein SNOG_04891 | GW996345 | 0.96±0.44 | **2.81±0.51** | 1.08±0.21 | 0.64±0.13 | 0.56±0.03 |
| Ar26 | Hypothetical protein | GW996349 | **2.51±0.10** | 0.85±0.25 | 1.32±0.61 | **2.37±0.55** | 1.72±0.10 |
| Ar29 | Hypothetical protein SNOG_07731 | GW996352 | 1.14±0.18 | **2.89±0.48** | 1.31±0.12 | 0.82±0.13 | 0.79±0.26 |
| Ar30 | Hypothetical protein | GW996353 | 1.26±0.05 | 1.69±0.28 | **2.96±0.26** | 0.89±0.19 | 1.37±0.02 |
| Ar32 | Zgc:158374 protein | GW996355 | 0.48±0.03 | 1.26±0.25 | 0.31±0.12 | 0.38±0.02 | 0.68±0.02 |
| Ar41 | Hypothetical protein SNOG_09961 | GW996364 | 1.12±0.37 | **3.16±0.73** | **2.29±0.06** | 0.80±0.13 | 1.28±0.47 |
| Ar45 | Hypothetical protein SNOG_03209 | GW996368 | 0.98±0.35 | **2.55±0.23** | 1.33±0.43 | 0.80±0.13 | 0.96±0.08 |
| Ar48 | Hypothetical protein SNOG_00366 | GW996371 | 1.70±0.20 | 0.91±0.33 | 0.91±0.16 | 1.17±0.18 | 1.45±0.18 |
| Ar51 | Hypothetical protein | GW996374 | 1.03±0.75 | **7.70±0.96** | **3.01±0.67** | 0.80±0.19 | 0.64±0.04 |
| Ar52 | Hypothetical protein SNOG_15982 | GW996375 | **6.26±0.61** | 1.04±0.37 | 1.28±0.30 | **4.38±0.72** | **2.59±0.21** |
| Ar54 | Hypothetical protein SNOG_00522 | GW996377 | 0.66±0.10 | 1.16±0.23 | 0.43±0.16 | 0.59±0.32 | 0.91±0.18 |
| Ar64 | Hypothetical protein | GW996387 | 1.94**±**0.20 | 1.21±0.46 | 0.64±0.05 | 0.70±0.34 | 0.40±0.39 |
| Ar66 | Hypothetical protein ACLA_073190 | GW996389 | 1.16±0.61 | 1.83±0.48 | 1.32±0.37 | 1.77±0.73 | 1.64±0.62 |
| Ar67 | Hypothetical protein SNOG_07177 | GW996390 | **2.60±0.71** | **2.77±0.51** | 1.16±0.47 | 1.38±0.55 | 1.32±0.70 |
| Ar69 | Hypothetical protein SNOG_10250 | GW996392 | **2.13±0.66** | 1.45±0.47 | 1.08±0.47 | 1.33±0.32 | 1.40±0.54 |
| Ar74 | Hypothetical protein | GW996397 | **4.24±0.70** | 1.68±0.49 | 1.36±0.34 | 1.69±0.38 | 1.64±0.35 |
| Ar78 | Hypothetical protein SNOG_00111 | GW996401 | **6.55±0.88** | **2.38±0.69** | 1.70±0.28 | **2.20±0.63** | **4.74±0.95** |
| Ar87 | Hypothetical protein NFIA_043490 | GW996410 | 0.54±0.17 | 0.79±0.27 | 0.23±0.08 | 0.170±0.06 | 0.22±0.09 |
| Ar88 | Hypothetical protein SNOG_12451 | GW996411 | 0.94±0.08 | 1.14±0.16 | 0.86±0.19 | 1.53±0.15 | 0.66±0.18 |
| Ar89 | Hypothetical protein SNOG_13725 | GW996412 | 1.61±0.16 | 1.10±0.15 | 0.95±0.26 | 1.22±0.01 | 0.73±0.02 |
| Ar92 | Hypothetical protein NFIA_061320 | GW996415 | 1.70±0.18 | 0.81±0.11 | 0.88±0.16 | 1.06±0.04 | 0.77±0.13 |
| Ar93 | Hypothetical protein AFUB_059580 | GW996416 | **22.45±1.83** | **2.39±0.43** | **5.31±0.57** | **8.61±0.61** | **5.79±0.46** |
| Ar97 | Hypothetical protein SNOG_12275 | GW996420 | **2.65±0.23** | 1.25±0.05 | 1.16±0.12 | **2.22±0.27** | 0.75±0.07 |
| Ar98 | Hypothetical protein SNOG_16005 | GW996421 | 1.53±0.08 | 1.00±0.04 | 0.78±0.06 | 1.25±0.07 | 0.76±0.07 |
| Ar100 | Conserved hypothetical protein | GW996423 | 1.45±0.15 | 1.49±0.00 | 1.33±0.17 | 1.69±0.25 | 0.66±0.13 |
| Ar101 | Conserved hypothetical protein | GW996424 | 1.06±0.32 | 1.74±0.26 | 1.03±0.15 | 1.62±0.37 | 0.77±0.03 |
| Ar105 | Hypothetical protein Bm1_07595 | GW996428 | **5.80±1.39** | 1.38±0.06 | 1.81±0.07 | **1.99±0.29** | **2.99±0.32** |
| Ar106 | Hypothetical protein SNOG_09955 | GW996429 | 1.80±0.12 | 0.86±0.38 | 0.80±0.05 | 0.84±0.06 | 0.54±0.04 |
| Ar113 | Conserved hypothetical protein | GW996436 | **7.88±0.31** | 1.22±0.02 | 1.60±0.10 | **2.06±0.06** | **2.20±0.09** |
| Ar116 | Hypothetical protein (BC1G_06909) | GW996439 | 0.89±0.16 | 1.09±0.59 | 0.89±0.20 | 0.81±0.27 | 0.18±0.05 |

^a^Clone_id assigned for each unigenes is given before their putative gene identity.

^b^BLASTX/tBLASTX searches were conducted to determine homologous genes and the putative function of the cDNA fragments.

^c^cDNA sequences of all unigenes listed in Table 1 have been submitted to the GenBank database and the assigned Accession numbers are mentioned.

^d^Ratios of signal intensity were determined by cDNA macroarray hybridization as described in the “Materials and methods”.

^e^Expression ratios of each gene are shown along with Standard Deviations (±SD) in response to menadione at 0.5h, 1h and 3h and also for H_2_O_2_ and NO response after 1h of treatment.

^f^Values are highlighted in bold if the expression ratios are more than 2-fold.
