# Supplementary material for: Comparative Transcriptome Analysis of the Necrotrophic Fungus Ascochyta rabiei during Oxidative Stress: Insight for Fungal Survival in the Host Plant
Source: PLoS One. 2012 Mar 12;7(3):e33128. doi: 10.1371/journal.pone.0033128 (PMC3299738; doi:10.1371/journal.pone.0033128)
Supplement: Table S2 — List of primers for real-time quantitative PCR. (DOC) [file pone.0033128.s008.doc]

Table S2: Primer sequences used for real time quantitative RT-PCR

| Gene identity | Symbol | Primers name | Primer sequence |
| --- | --- | --- | --- |
| Hypothetical protein SNOG_16463 |  | Ar2RT1F  Ar2RT1R | 5’ CGGAGACAGTAGTGAGGATGAACA 3’  5’ CGATACCGGATGTGCAGATTG 3’ |
| Cytochrome C | CYC | Ar3RT1F Ar3RT1R | 5’ CATCACTATCACCAATCCCTACCA 3’  5’ AGCATCGCCCTTCTCGTAAC 3’ |
| E3 SUMO-protein ligase PIAS1 | PIAS1 | Ar9RTF  Ar9RTR | 5’ GACCTGACGTTGAGCGATGA 3’  5’ TCTGCCCTTGCCTTTTTAGC 3’ |
| Carotenoid oxygenase | COX | Ar11RTF  Ar11RTR | 5’ AAATTCGGAATGCGCTTGTC 3’  5’ GCCTCGCACAATGCCTTT 3’ |
| Acetylglutamate kinase | ACT | Ar12RTF  Ar12RTR | 5’ CGCTGTTTGGTTCCAGGGTAT 3’  5’ CGGCGTAGCGGTCTTGATA 3’ |
| F-box and WD domain containing protein | FBO | Ar13RTF  Ar13RTR | 5’ TTGTCTCGGCTGGGACATG 3’  5’ TCATCGCCAGCGAAATCA 3’ |
| ATP-citrate synthase | ACS | Ar14RTF  Ar14RTR | 5’ GGTGGTGTTGACGTTGGTGAT 3’  5’ TCGACGGGAATGAGAAGCTT 3’ |
| Thioredoxin | TRX | Ar15RTF  Ar15RTR | 5’ CCCGATGTCGCACAAGAACT 3’  5’ ACCTCGGCGACCTTGTCA 3’ |
| FMN dependent dehydrogenase | FMN | Ar19RTF  Ar19RTR | 5’ GCGCGAGAACGCATGTC 3’  5’ TCCTGCATTGCCAATCACA 3’ |
| Neutral trehalase | TRE | Ar25RTF  Ar25RTR | 5’ GCCTCGCGAAGGTTTCG 3’  5’ AAGCGTCATGCCGTAGATGTAA 3’ |
| Hypothetical protein |  | Ar26RT1F  Ar26RT1R | 5’ GCCCACCTGATACGGAGATTC 3’  5’ GTTTCCTCGGGCTAGCTATGAC 3’ |
| NADH oxidase | NOX | Ar34RT1F Ar34RT1R | 5’ GCCACCGACGAGGACATT 3’  5’ CTGGGCTGCGTGGACAA 3’ |
| Catalase | CAT | Ar35RTF  Ar35RTR | 5’ GCTCATCGAAACGCTTGCA 3’  5’ TGCACGACTCGCTCAGGTAT 3’ |
| C6 transcription factor | C6 | Ar36RTF  Ar36RTR | 5’ CCTCGGACCGCTGTTCACT 3’  5’ GCAGCCCAACCTTCAAGATC 3’ |
| Heat shock protein SSC1-like protein | SSC1 | Ar38RTF  Ar38RTR | 5’ AAGACCGACGAGCTCCAGAA 3’  5’ GCGGGCCTTGTGCATCT 3’ |
| Ubiquitin-conjugating enzyme E2 N | E2N | Ar46RTF  Ar46RTR | 5’ CGTTTTGGAAGGGATGCAA 3’  5’ TCGGGCAGGCGTGACT 3’ |
| Hypothetical protein SNOG_00366 |  | Ar48RTF  Ar48RTR | 5’ GGGCAAGGACGGCTACTAGA 3’  5’ TGGTGTCTGCTCTTCTCAAAGC 3’ |
| Dna k-type molecular chaperone BiP | BIP | Ar49RTF  Ar49RTR | 5’ AGGACGCTAAGACCATGGGTAA 3’  5’ AGAGTGCGCTTGGCCTTCT 3’ |
| 41 kDa peptidyl-prolyl cis-trans isomerase | PPI | Ar50RTF  Ar50RTR | 5’ CGCCGTATTGCCCATGTC 3’  5’ GGCGCACAGATCCTGAAGA 3’ |
| Ribosomal protein S5 | RPS5 | Ar55RTF  Ar55RTR | 5’ GCCACCTTCGTCGCTGTT 3’  5’ GTCCACAGGTCGGGTGTGA 3’ |
| Mannosylphosphate transferase (Mnn4) | MNN4 | Ar57RTF  Ar57RTR | 5’ CAGCGCGCGTCTATGATATTC 3’  5’ ATCCTTGGATCTGGCAACGT 3’ |
| Glyceraldehyde 3-phosphate dehydrogenase | GPDAH | Ar61RTF  Ar61RTR | 5’ GCGCTCAAAGCTTAGGCATAG 3’  5’ GGTTGATGGCAACGCTTAGG 3’ |
| NADH-ubiquinone oxidoreductase | NUO | Ar65RT1F Ar65RT1R | 5’ CCGCCACATCAACATTCCTA 3’  5’ GGCCACCAGAGGAGCTTGT 3’ |
| Hypothetical protein ACLA_073190 |  | Ar66RTF  Ar66RTR | 5’ TCTCGGCGCCATGGTCTA 3’  5’ ACAAGCAAATGACCAACGAGTTT 3’ |
| Hypothetical protein SNOG_10250 |  | Ar69RTF  Ar69RTR | 5’ GGTGGGAGGCGTCTGTATTG 3’  5’ CGGCCTGCCCTAATCACA 3’ |
| Ubiquitin |  | Ar71RTF  Ar71RTR | 5’ AAACCAAGACCCCAGCTCGTA 3’  5’ GGGCCGATGATGGTTTCTG 3’ |
| Hypothetical protein |  | Ar74RT1F  Ar74RT1R | 5’ AGAGTCGATTGCGAGGAGGTT 3’  5’ GCAACGCAATTCCAAACATG 3’ |
| C2 domain containing protein |  | Ar77RT1F  Ar77RT1R | 5’ CGGCGGCACAAAAGAAAT 3’  5’ CTGTGATACGAGCCTCTGTTTGTT 3’ |
| Alternative oxidase | AOX | Ar81UTRTF  Ar81UTRTR | 5’ GTCGTCATCCTACAGCATTTCCT 3’  5’ AAAACGCCGCGGAACA 3’ |
| Protein phosphatase 2A | PP2A | Ar86RTF  Ar86RTR | 5’ TGGTCTGCTTGAAGCTTCGA 3’  5’ TGGTTGCCGCGAATGAG 3’ |
| Plasma membrane ATPase |  | Ar96RTF  Ar96RTR | 5’ TCTTCCTGTGGCGGTCCTT 3’  5’ TCTACTACCTTCTCAACCGCATTG 3’ |
| Usp domain-containing |  | Ar104RTF  Ar104RTR | 5’ TCGCAGTCGTTTCCTAGCAA 3’  5’ CAGCAACTACCTCGTCACCAAGT 3’ |
| Elongation factor 1α | Ef1α | ArEFUTRF  ArEFUTRR | 5’ GATCACTTTTTCGGTCGTTTGTT 3’  5’ CTTCGTTCCACCAGACCGTAA 3’ |
