# Supplementary material for: Comparative Transcriptome Analysis of the Necrotrophic Fungus Ascochyta rabiei during Oxidative Stress: Insight for Fungal Survival in the Host Plant
Source: PLoS One. 2012 Mar 12;7(3):e33128. doi: 10.1371/journal.pone.0033128 (PMC3299738; doi:10.1371/journal.pone.0033128)
Supplement: Table S3 — Group of clusters after SOTA analysis. (DOC) [file pone.0033128.s009.doc]

Table S3: SOTA clustering performed on expression ratios obtained by macroarray analysis after menadione, H_2_O_2_ and NO treatments. Detailed information of each cluster is given here.

| Original row | Accession No. | CLONE_ID | DESC | Menadione | H_2_O_2_ | NO |
| --- | --- | --- | --- | --- | --- | --- |
| Cluster1 |  |  |  |  |  |  |
| 45 | GW996369 | Ar46 | Ubiquitin-conjugating enzyme E2 N | 2.630057 | 1.014466 | 1.3677157 |
| 60 | GW996384 | Ar61 | Glyceraldehyde 3-phosphate dehydrogenase | 1.809029 | 0.480079 | 1.892663 |
| 71 | GW996395 | Ar72 | Protein kinase C conserved region 1 (C1) | 1.552449 | 1.066138 | 1.0193888 |
| 78 | GW996402 | Ar79 | Endosomal cargo receptor (Erv14) | 1.31174 | 0.823617 | 1.2255564 |
| 74 | GW996398 | Ar75 | MARVEL, Membrane-associating domain | 1.298508 | 0.989476 | 1.0146949 |
| 77 | GW996401 | Ar78 | Hypothetical protein SNOG_00111 | 1.251057 | 1.14154 | 2.245023 |
|  |  |  |  |  |  |  |
| Cluster 2 |  |  |  |  |  |  |
| 42 | GW996366 | Ar43 | PhiA protein | 1.546046 | 1.957798 | 2.2395847 |
| 70 | GW996394 | Ar71 | Ubiquitin | 2.276634 | 1.93167 | 2.6158588 |
| 83 | GW996408 | Ar85 | GTP-binding protein EsdC | 1.755065 | 2.012439 | 2.590246 |
| 90 | GW996416 | Ar93 | Hypothetical protein AFUB_059580 | 1.262998 | 3.10636 | 2.5348198 |
|  |  |  |  |  |  |  |
| Cluster 3 |  |  |  |  |  |  |
| 34 | GW996358 | Ar35 | Catalase | 1.897807 | 2.354501 | 0.6202236 |
| 58 | GW996382 | Ar59 | 30 kDa heat shock protein | 1.35444 | 1.798042 | 1.858522 |
|  |  |  |  |  |  |  |
| Cluster 4 |  |  |  |  |  |  |
| 10 | GW996333 | Ar10 | Hypothetical protein PTRG_10228 | 1.391802 | 0.545086 | 1.2801307 |
| 13 | GW996336 | Ar13 | F-box and WD domain containing protein | 1.598829 | 0.376912 | 0.2928245 |
| 17 | GW996340 | Ar17 | Cell surface protein, putative | 2.651186 | 1.063466 | 0.8531534 |
| 18 | GW996342 | Ar19 | FMN dependent dehydrogenase | 1.536659 | -0.10232 | 0.7781417 |
| 37 | GW996361 | Ar38 | Heat shock protein SSC1-like protein | 2.280448 | -0.02744 | 0.7408705 |
| 38 | GW996362 | Ar39 | Ribosomal protein L26 | 2.553021 | 0.012398 | 0.4842385 |
| 39 | GW996363 | Ar40 | 60S acidic ribosomal protein P0 | 1.711512 | -0.13275 | 0.7249891 |
| 46 | GW996370 | Ar47 | Hypothetical protein | 2.987055 | -0.12138 | 1.2592922 |
| 48 | GW996372 | Ar49 | dnaK-type molecular chaperone BiP | 1.975008 | 0.603104 | -0.038802 |
| 49 | GW996373 | Ar50 | 41 kDa peptidyl-prolyl cis-trans isomerase | 2.432903 | 0.658268 | 0.4486919 |
| 56 | GW996380 | Ar57 | Mannosylphosphate transferase (Mnn4), putative | 0.91367 | -0.39986 | 1.037591 |
| 66 | GW996390 | Ar67 | Hypothetical protein SNOG_07177 | 1.470257 | 0.474292 | 0.4037826 |
| 79 | GW996403 | Ar80 | Cell lysis protein-like | 1.531276 | 0.837955 | 0.1344639 |
| 40 | GW996364 | Ar41 | Hypothetical protein SNOG_09961 | 1.661874 | -0.31609 | 0.3654884 |
|  |  |  |  |  |  |  |
| Cluster 5 |  |  |  |  |  |  |
| 15 | GW996338 | Ar15 | TRX_family | 0.591131 | 1.973851 | 1.2285061 |
| 51 | GW996375 | Ar52 | Hypothetical protein SNOG_15982 | 0.058725 | 2.133414 | 1.3753438 |
| 54 | GW996378 | Ar55 | Ribosomal protein S5 | 0.115298 | 1.809868 | 2.0124958 |
| 72 | GW996396 | Ar73 | RING-8 protein | 0.911992 | 1.524422 | 0.8271956 |
| 82 | GW996407 | Ar84 | Multidrug resistant protein | 0.038299 | 1.556575 | 0.6520971 |
| 101 | GW996428 | Ar105 | Hypothetical protein Bm1_07595 | 0.472624 | 0.993868 | 1.5824838 |
| 104 | GW996436 | Ar113 | Conserved hypothetical protein | 0.29598 | 1.049439 | 1.1390029 |
|  |  |  |  |  |  |  |
| Cluster 6 |  |  |  |  |  |  |
| 2 | GW996325 | Ar2 | Hypothetical protein SNOG_16463 | 0.984936 | 0.920962 | 0.1637217 |
| 3 | GW996326 | Ar3 | Cytochrome c | 0.379835 | 0.472552 | 0.7258498 |
| 11 | GW996334 | Ar11 | Cartenoid oxygenase, putative | -0.058609 | 1.32042 | 0.0431164 |
| 12 | GW996335 | Ar12 | Acetylglutamate kinase, putative | 0.560661 | 1.324005 | -0.095192 |
| 23 | GW996347 | Ar24 | Ribonucleoside-diphosphate reductase subunit M2 | 0.457633 | 0.250557 | 1.1541557 |
| 24 | GW996348 | Ar25 | Neutral trehalase | 0.096122 | 0.292798 | 0.7155495 |
| 25 | GW996349 | Ar26 | Hypothetical protein | -0.233778 | 1.249164 | 0.787838 |
| 65 | GW996389 | Ar66 | Conserved hypothetical protein (ACLA_073190 | 0.876336 | 0.829812 | 0.7188554 |
| 68 | GW996392 | Ar69 | Hypothetical protein SNOG_10250 | 0.536932 | 0.411544 | 0.4951221 |
| 73 | GW996397 | Ar74 | Hypothetical protein | 0.755632 | 0.757282 | 0.7153378 |
| 76 | GW996400 | Ar77 | C2, Protein kinase C conserved region 2 (CalB) | 0.30442 | 1.058499 | 0.3810546 |
| 80 | GW996404 | Ar81 | Alternative oxidase, mitochondrial precursor | 1.076891 | 0.786607 | 1.058032 |
| 91 | GW996417 | Ar94 | Superoxide dismutase, mitochondrial precursor | 1.04021 | 0.553564 | 0.3824306 |
| 92 | GW996418 | Ar95 | Myo-inositol-phosphate synthase, putative | 0.455901 | 1.00082 | 0.022611 |
| 93 | GW996419 | Ar96 | Plasma membrane ATPase | 0.84676 | 1.548663 | 0.2338347 |
| 14 | GW996337 | Ar14 | ATP-citrate synthase | 0.331583 | 1.714769 | -0.724615 |
| 47 | GW996371 | Ar48 | Hypothetical protein SNOG_00366 | -0.130356 | 0.235718 | 0.536729 |
| 64 | GW996388 | Ar65 | NADH-ubiquinone oxidoreductase 39 kDa subunit, putative | 0.323887 | 0.67728 | -0.064821 |
| 94 | GW996420 | Ar97 | Hypothetical protein SNOG_12275 | 0.331256 | 1.152502 | -0.399915 |
|  |  |  |  |  |  |  |
| Cluster 7 |  |  |  |  |  |  |
| 85 | GW996410 | Ar87 | Hypothetical protein NFIA_043490 | -0.335936 | -2.55399 | -2.173231 |
|  |  |  |  |  |  |  |
| Cluster 8 |  |  |  |  |  |  |
| 22 | GW996346 | Ar23 | ChtBD1, Chitin binding domain | -0.875779 | -0.78932 | -1.044174 |
|  |  |  |  |  |  |  |
| Cluster 9 |  |  |  |  |  |  |
| 19 | GW996343 | Ar20 | Phosphoinositide 3-phosphate phosphatase | -0.093342 | -0.30771 | -0.011535 |
| 32 | GW996356 | Ar33 | Zn cluster transcription factor Rds2, putative | 0.212092 | -0.22354 | 0.5134547 |
| 43 | GW996367 | Ar44 | Acriflavin resistance protein | -0.094769 | -1.1315 | 0.3446046 |
| 52 | GW996376 | Ar53 | Phospho-2-dehydro-3-deoxyheptonate aldolase | -0.056975 | -0.44195 | 0.0097147 |
| 53 | GW996377 | Ar54 | Hypothetical protein SNOG_00522 | 0.224429 | -0.75306 | -0.121273 |
| 31 | GW996355 | Ar32 | Zgc:158374 protein | 0.333919 | -1.39102 | -0.550413 |
|  |  |  |  |  |  |  |
| Cluster 10 |  |  |  |  |  |  |
| 5 | GW996328 | Ar5 | DUF1741 family protein | -0.035256 | 0.053232 | -1.176234 |
| 7 | GW996330 | Ar7 | Uracil phosphoribosyltransferase | 0.033067 | 0.417482 | -0.720069 |
| 8 | GW996331 | Ar8 | Histone H1 | 0.482617 | 0.672508 | -0.466152 |
| 63 | GW996387 | Ar64 | Hypothetical protein | 0.283911 | -0.49998 | -1.28885 |
| 69 | GW996393 | Ar70 | RAC-alpha serine/threonine-protein kinase | 0.334465 | -0.0953 | -0.394899 |
| 86 | GW996411 | Ar88 | Hypothetical protein SNOG_12451 | 0.192746 | 0.616893 | -0.596964 |
| 87 | GW996412 | Ar89 | Hypothetical protein SNOG_13725 | 0.148768 | 0.292696 | -0.443251 |
| 89 | GW996415 | Ar92 | Hypothetical protein NFIA_061320 | -0.295143 | 0.094278 | -0.376337 |
| 95 | GW996421 | Ar98 | Hypothetical protein SNOG_16005 | 9.45E-04 | 0.329393 | -0.381345 |
| 96 | GW996423 | Ar100 | Conserved hypothetical protein | 0.578544 | 0.764857 | -0.591877 |
| 98 | GW996425 | Ar102 | Molybdopterin binding domain protein | 0.108233 | 0.018898 | -0.718526 |
| 100 | GW996427 | Ar104 | Usp domain-containing protein | 0.369674 | -0.10824 | -1.594803 |
| 103 | GW996435 | Ar112 | Large subunit ribosomal RNA gene | 0.217602 | 0.374042 | -0.428334 |
| 105 | GW996439 | Ar116 | Hypothetical protein (BC1G_06909) | 0.129073 | -0.29321 | -2.401817 |
| 106 | GW996442 | Ar119 | Nucleosome assembly protein | -0.201357 | 0.05426 | -0.382045 |
| 102 | GW996429 | Ar106 | Hypothetical protein SNOG_09955 | -0.210114 | -0.24282 | -0.869944 |
|  |  |  |  |  |  |  |
| Cluster 11 |  |  |  |  |  |  |
| 1 | GW996324 | Ar1 | Export control protein CHS7-like, putative | 0.997012 | 0.064922 | -0.708516 |
| 4 | GW996327 | Ar4 | 60S ribosomal protein P0 | 2.082118 | -0.11088 | -1.316868 |
| 6 | GW996329 | Ar6 | 3-hydroxyisobutyryl-CoA hydrolase, mitochondrial precursor | 1.903194 | 0.055608 | -0.995277 |
| 9 | GW996332 | Ar9 | E3 SUMO-protein ligase PIAS1 | 1.027461 | 0.917571 | -0.219651 |
| 16 | GW996339 | Ar16 | Phosphoserine phosphatase, putative | 0.730005 | 0.138197 | -0.210235 |
| 20 | GW996344 | Ar21 | Hypothetical protein SNOG_10728 | 1.284747 | 0.361905 | -0.336854 |
| 21 | GW996345 | Ar22 | Hypothetical protein SNOG_04891 | 1.49092 | -0.62385 | -0.83328 |
| 26 | GW996350 | Ar27 | SecE/Sec61-gamma subunits of protein translocation complex | 0.670048 | 0.099765 | 0.0095302 |
| 27 | GW996351 | Ar28 | Fatty acid synthase subunit beta dehydratase | 1.012971 | -0.49739 | -0.18001 |
| 28 | GW996352 | Ar29 | Hypothetical protein SNOG_07731 | 1.534247 | -0.27654 | -0.322282 |
| 29 | GW996353 | Ar30 | Hypothetical protein | 0.762185 | -0.15407 | 0.4638556 |
| 30 | GW996354 | Ar31 | Zinc knuckle transcription factor/splicing factor MSL5/ZFM1 | 1.023989 | -0.6401 | 0.0184048 |
| 33 | GW996357 | Ar34 | NADH oxidase | 1.158482 | 0.018572 | 0.4105131 |
| 35 | GW996359 | Ar36 | C6 transcription factor, putative | 1.013011 | -0.18898 | 0.450123 |
| 36 | GW996360 | Ar37 | Acyl-CoA desaturase | 1.300614 | -0.34993 | -0.985268 |
| 41 | GW996365 | Ar42 | pre-rRNA-processing protein TSR2 | 1.417208 | -0.48765 | -0.26603 |
| 44 | GW996368 | Ar45 | Hypothetical protein SNOG_03209 | 1.352044 | -0.30972 | -0.055592 |
| 50 | GW996374 | Ar51 | Hypothetical protein | 2.944912 | -0.31133 | -0.625972 |
| 55 | GW996379 | Ar56 | Ribosomal protein S5 | 1.05314 | -0.42637 | 0.390577 |
| 57 | GW996381 | Ar58 | Mitochondria fission 1 protein | 1.627018 | -0.48479 | -0.148441 |
| 59 | GW996383 | Ar60 | RNA polymerase II subunit | 0.946715 | -0.26808 | -0.482927 |
| 61 | GW996385 | Ar62 | niemann-Pick C1 protein precursor | 1.447879 | 0.432008 | -1.553496 |
| 62 | GW996386 | Ar63 | Heat shock protein 78, mitochondrial precursor | 0.696791 | 0.318473 | -0.162226 |
| 67 | GW996391 | Ar68 | 60S ribosomal protein L36 | 1.390721 | 0.786561 | -0.801823 |
| 75 | GW996399 | Ar76 | Mitochondrial phosphate carrier protein | 1.277755 | 0.131019 | 0.0797118 |
| 81 | GW996406 | Ar83 | Hypothetical protein SNOG_09667 | 1.393103 | -0.43533 | -0.616664 |
| 84 | GW996409 | Ar86 | Serine/threonine-protein phosphatase PP2A catalytic subunit | 1.342849 | 0.05775 | -0.335343 |
| 88 | GW996413 | Ar90 | Annexin A7 | 0.875175 | -0.24133 | -0.097274 |
| 97 | GW996424 | Ar101 | Hypothetical protein SNOG_03050 | 0.80644 | 0.704658 | -0.360143 |
| 99 | GW996426 | Ar103 | Hypothetical protein SNOG_01225 | 1.353393 | -0.74324 | -1.503785 |
