# Supplementary material for: Comparative Transcriptome Analysis of the Necrotrophic Fungus Ascochyta rabiei during Oxidative Stress: Insight for Fungal Survival in the Host Plant
Source: PLoS One. 2012 Mar 12;7(3):e33128. doi: 10.1371/journal.pone.0033128 (PMC3299738; doi:10.1371/journal.pone.0033128)
Supplement: Table S4 — Relative expression of selected genes in planta determined by qRT-PCR. (DOC) [file pone.0033128.s010.doc]

Table S4: Relative expression of selected genes *in planta* by using qRT-PCR.

| **Clone ID** | **Gene name** | **1d** | **3d** | **6d** |
| --- | --- | --- | --- | --- |
| **Genes of cluster 6** |  |  |  |  |
| *Ar2* | Hypothetical protein SNOG_16463 | 6.57±0.51 | 2.56±0.39 | 8.67±0.65 |
| *Ar3* | Cytochrome C | 3.03±0.52 | 1.39±0.13 | 1.42±0.27 |
| *Ar11* | Carotenoid oxygenase | 1.52±0.12 | 3.90±0.44 | 10.31±0.06 |
| *Ar12* | Acetylglutamate kinase | 9.74±2.39 | 289.22±58.66 | 576.88±54.53 |
| *Ar25* | Neutral trehalase | 0.83±0.24 | 0.73±0.04 | 1.38±0.16 |
| *Ar26* | Hypothetical protein | 5.10±0.58 | 3.77±0.69 | 11.15±1.03 |
| *Ar66* | Hypothetical protein ACLA_073190 | 3.02±0.36 | 33.34±5.86 | 13.32±4.15 |
| *Ar69* | Hypothetical protein SNOG_10250 | 3.33±0.67 | 3.08±0.71 | 1.22±0.12 |
| *Ar74* | Hypothetical protein | 0.24±0.04 | 0.25±0.02 | 0.10±0.01 |
| *Ar77* | C2 domain containing protein | 0.13±0.02 | 0.35±0.07 | 0.70±0.13 |
| *Ar81* | Alternative oxidase | 0.20±0.01 | 0.14±0.01 | 0.27±0.03 |
| *Ar96* | Plasma membrane ATPase | 1.07±0.06 | 0.50±0.13 | 0.74±0.13 |
| *Ar14* | ATP-citrate synthase | 0.53±0.08 | 0.43±0.07 | 0.46±0.03 |
| *Ar48* | Hypothetical protein SNOG_00366 | 0.65±0.05 | 0.16±0.02 | 0.45±0.07 |
| *Ar65* | NADH-ubiquinone oxidoreductase | 0.44±0.09 | 2.18±0.32 | 45.68±7.99 |
| **Stress responsive genes** |  |  |  |  |
| *Ar19* | FMN dependent dehydrogenase | 0.16±0.01 | 0.22±0.01 | 0.14±0.06 |
| *Ar34* | NADH oxidase | 3.19±0.22 | 4.03±0.62 | 15.44±1.78 |
| *Ar35* | Catalase | 11.40±1.03 | 5.61±0.50 | 42.19±0.60 |
| *Ar15* | Thioredoxin | 0.19±0.01 | 1.25±0.06 | 0.70±0.04 |
| *Ar9* | E3 SUMO-protein ligase PIAS1 | 4.22±0.88 | 53.04±8.42 | 39.55±9.63 |
| *Ar13* | F-box and WD domain containing protein | 180.69±29.87 | 68.57±17.10 | 136.44±8.53 |
| *Ar46* | Ubiquitin-conjugating enzyme E2 N | 0.25±0.01 | 0.78±0.03 | 1.04±0.08 |
| *Ar71* | Ubiquitin | 0.31±0.01 | 0.07±0.01 | 0.08±0.01 |
| *Ar104* | Usp domain-containing protein | 30.81±2.47 | 7.20±0.93 | 10.68±1.82 |
| *Ar57* | Mannosylphosphate transferase (Mnn4) | 0.58±0.08 | 0.24±0.04 | 0.58±0.01 |
| **Other genes** |  |  |  |  |
| Ar36 | C6 transcription factor | 0.29±1.56 | 2.80±0.39 | 10.84±1.00 |
| Ar49 | Molecular chaperone BiP | 8.62±1.29 | 6.06±1.84 | 266.84±0.14 |
| Ar50 | peptidyl-prolyl cis-trans isomerase | 20.52±1.09 | 36.82±0.19 | 2.58±5.51 |
| Ar55 | Ribosomal protein S5 | 12.13±0.05 | 2.39±0.66 | 3.32±2.55 |
| Ar86 | protein phosphatase PP2A | 355.66±11.93 | 218.51±19.73 | 221.79±9.06 |
